# Receptor-Targeted Carbon Nanodot Delivery through Polymer Caging and Click Chemistry-Supported LRP1 Ligand Attachment

**DOI:** 10.3390/polym15204039

**Published:** 2023-10-10

**Authors:** Fengrong Zhang, Teoman Benli-Hoppe, Wei Guo, Johanna Seidl, Yi Wang, Rongqin Huang, Ernst Wagner

**Affiliations:** 1 Pharmaceutical Biotechnology, Department of Pharmacy, Center for NanoScience, Ludwig-Maximilians-Universität Munich, 81377 Munich, Germany; fengrong.zhang@cup.uni-muenchen.de (F.Z.); teoman.benli-hoppe@cup.uni-muenchen.de (T.B.-H.); johanna.seidl@cup.uni-muenchen.de (J.S.); 2 Department of Pharmaceutics, School of Pharmacy, Key Laboratory of Smart Drug Delivery, Ministry of Education, Fudan University, Shanghai 201203, China; 20111030047@fudan.edu.cn; 3 Center for Advanced Low-dimension Materials, State Key Laboratory for Modification of Chemical Fibers and Polymer Materials, College of Chemistry, Chemical Engineering and Biotechnology, Donghua University, Shanghai 201620, China; ywang@dhu.edu.cn

**Keywords:** click chemistry, retro-enantio ligand, low-density lipoprotein receptor, carbon nanodot, sequence-defined oligomer

## Abstract

Carbon nanodots present resistance to photobleaching, bright photoluminescence, and superior biocompatibility, making them highly promising for bioimaging applications. Herein, nanoprobes were caged with four-armed oligomers and subsequently modified with a novel DBCO–PEG-modified retro-enantio peptide ligand reL57, enhancing cellular uptake into U87MG glioma cells highly expressing low-density lipoprotein receptor-related protein 1 (LRP1). A key point in the development of the oligomers was the incorporation of ε-amino-linked lysines instead of standard α-amino-linked lysines, which considerably extended the contour length per monomer. The four-armed oligomer ***1696*** was identified as the best performer, spanning a contour length of ~8.42 nm for each arm, and was based on an altering motive of two cationic ε-amidated lysine tripeptides and two tyrosine tripeptides for electrostatic and aromatic stabilization of the resulting formulations, cysteines for disulfide-based caging, and N-terminal azidolysines for click-modification. This work highlights that well-designed four-armed oligomers can be used for noncovalent coating and covalent caging of nanoprobes, and click modification using a novel LRP1-directed peptide ligand facilitates delivery into receptor-expressing target cells.

## 1. Introduction

Early detection of numerous diseases, particularly glioblastoma, the most aggressive brain tumor, is a critical aspect in clinical practice; with the help of diagnostics, the five-year survival rate for malignant brain and other central nervous system tumors in the United States has risen to 35.7% [1,2]. Unlike traditional organic small molecules that possess deficiencies such as poor solubility as well as photo-instability [3,4], carbon nanodots (zero-dimensional nanoparticles) with a sp^2^- and sp^3^-hybridized carbon skeleton offer resistance to photobleaching and superior aqueous dispersibility, making them new contributors to medical imaging [5,6,7,8]. Importantly, the emission wavelength of carbon nanodots can be well-regulated into the near-infrared or red region through heteroatom-doping and creating surface defects [9,10,11,12]. The deeper tissue penetration of red carbon nanodots (RCDs) is therefore well achievable based on long-wavelength emission [13,14,15].

Nevertheless, two distinguishing marks of clinical importance that characterize brain tumors are that they have restricted vascular barriers as well as a dense extracellular matrix with poor permeability. These two features, defined as the physiological and pathological barriers, respectively, pose technological bottlenecks for the delivery of nanoprobes to the pathological site [16,17]. Additionally, naked ultrasmall carbon nanodots (less than 5.5 nm in size) typically undergo rapid clearance from the bloodstream through the renal removal route, so attenuating the effective accumulation and inducing function loss [18,19]. Prolonged systemic circulation and efficient biodegradation often present conflicting requirements for most nanoparticles, further complicating this issue.

Enhancing the permeability of nanoprobes into the brain via receptor-mediated transcytosis across the blood–brain barrier (BBB) results in high specificity, and is, therefore, a strategy that is extensively investigated [20,21,22]. Low-density lipoprotein receptor-related protein 1 (LRP1) is a large single-pass transmembrane receptor that is highly expressed in brain endothelial cells and upregulated in some effector cells, such as melanized neurons in the substantia nigra and glioma cells [23,24,25]. Ligands out of this receptor family, including apolipoprotein E, lactoferrin, and angiopep-2, have been well explored [2,26,27,28]. Angiopep-2, a 19-amino-acid long ligand, is of particular interest; it has been reported to effectively cross the BBB and deliver covalently bound drugs into the brain [2,29]. Building on the success of angiopep-2, the sequence of this ligand was used as a basis for investigating potential enhancements, leading to the synthesis of L57 (sequence: TWPKHFDKHTFYSILKLGKH–COOH) as a novel artificial LRP1 ligand with superior performance in cellular uptake and BBB permeability compared to angiopep-2 [30].

In the aforementioned targeting strategies, reliable methods for coating drug nanoparticles are of utmost importance. Strategies for covalent modifications, encapsulation within lipid membranes, and noncovalent surface modifications of nanoparticles have been pursued. A series of physicochemical strategies to enhance targeted drug delivery by polymeric micelles have been reported [31,32]. These approaches include smart processes such as the physical complexation of macromolecules followed by subsequent covalent entrapment [33]. In our previous work, a noncovalent coating method for protein nanoparticle using cationic four-arm oligomers was developed, which mediated intracellular nanobody delivery in a receptor-facilitated process [34]. The presence of disulfide-forming cysteines in the four-armed oligomers was found to be crucial for the formation of stable protein nanoparticles. In our current work, a new library of four-armed oligomers was established to enable surface shielding and targeting of RCDs. These oligomers were synthesized by a solid-phase supported peptide synthesis (SPPS) methodology analogously as in our own and other previous work [35,36,37]. To achieve successful layer coating of the negatively charged hydroxynaphthalene-derived RCDs through electrostatic interactions, each of the oligomers should contain two or three lysine tripeptide motives with positively charged amines. The lysine units were incorporated as standard α-amino or ε-amino amidated lysine amides. Optionally, alternating blocks of tyrosine or leucine tripeptide were included, serving either as aromatic or hydrophobic domains to further stabilize the coating of RCDs. At the N-terminus of each arm, cysteines and azidolysines were included. Cysteines were considered to form stabilizing disulfide bonds to enhance the caging of the RCDs. The azido moieties were introduced for subsequent strain-promoted azide-alkyne cycloaddition (SPAAC), to enable shielding and targeting of caged RCDs with ligand–PEG–dibenzocyclooctyne (DBCO) conjugates. Within the current project, a novel protease-resistant retro-enantio peptide analog of L57 (reL57) was also developed and characterized.

## 2. Materials and Methods

### 2.1. Materials and Chemicals

Fmoc–Ala–Wang resin, 2-chlorotrityl resin, and protected Fmoc–amino acids were acquired from Iris Biotech (Marktredewitz, Germany). Triton™ X-100, 1-hydroxybenzotriazole (HOBt), acetic anhydride, 1,2-ethanedithiol (EDT), triisopropylsilane (TIS), *N,N,N*′*,N*′-tetramethyl-*O-*(1*H*-benzotriazol-1-yl)uronium hexafluorophosphate (HBTU), 1,8-diazabicyclo undec-7-ene (DBU), tris(2-carboxyethyl)phosphine (TCEP), (benzotriazol-1-yloxy) tripyrrolidino phosphonium hexafluorophosphate (PyBOP), *N*,*N*-diisopropylethylamine (DIPEA), *N*-methyl-2-pyrrolidone (NMP), and methyl thiazolyl tetrazolium (MTT) were sourced from Sigma-Aldrich (Munich, Germany). *N*-(2-hydroxyethyl)piperazine-*N*′-(2-ethanesulfonic acid) (HEPES) was purchased from Biomol GmbH (Hamburg, Germany). *N*-hexane and *tert*-butyl methyl ether (MTBE) were obtained from Brenntag GmbH (Essen, Germany). Fmoc–*N*-amido–dPEG_24_–acid was supplied by Quanta Biodesign (Plain City, OH, USA). Trifluoroacetic acid (TFA) and Ellman’s reagent were purchased from Thermo Scientific (Waltham, MA, USA). Cell culture media, penicillin, fetal bovine serum (FBS), and streptomycin were obtained from Invitrogen (Karlsruhe, Germany). Endothelial Cell Growth Medium 2 and Growth Medium SupplementMix were procured from PromoCell (Heidelberg, Germany). Syringe microreactors for ligands synthesis were obtained from MultiSynTech (Witten, Germany). The protected amino acids used in peptide synthesis are listed in Table S1 in the Supplementary Materials. All chemicals mentioned were utilized as received without further treatment. Further details can be found in the original Ph.D. thesis of T. B.-H. [38].

### 2.2. OAA Synthesis

With the assistance of Fmoc-based SPPS, four-armed oligomers ***1658***, ***1664***, ***1696***, ***1768***, and ***1769***, containing N-terminal azidolysines, were synthesized in syringe reactors. Fmoc–Ala–Wang resin served as solid support. The synthesis of the four-armed structures involved three main processes: loading of Fmoc–Lys(Fmoc)–OH to the resin, coupling of Fmoc–amino acids to extend the sequence, and cleaving of the resin. In detail, the backbone of the oligomer was automatically synthesized by a peptide synthesizer (Biotage, Uppsala, Sweden). Reagents were prepared as follows: 4 equivalents (eq) of HOBt were dissolved in DMF with 1% Triton™ X-100, 4 eq of Fmoc-protected amino acid were dissolved in the solution; 4 eq of HBTU were dissolved in DMF with 1% Triton™ X-100, and 8 eq of DIPEA were dissolved in NMP containing 1% Triton™ X-100. Every coupling reaction was carried out two times for 12 min at 75 °C. Manual couplings were performed as single couplings with a 120 min incubation period. All couplings that involved cysteine were conducted at room temperature (RT) to prevent cross-linking of free thiols. After coupling, deprotection was performed in 4 cycles for 12 min with a DMF solution containing 1% Triton™ X-100, 2% DBU, and 20% piperidine. After coupling and deprotection, the resin was washed with DMF 5 times. The introduction of branching points within the oligomer backbone was realized by utilizing Fmoc–Lys(Fmoc)–OH. After completing the entire oligomer sequences, the resins were vacuum-dried before cleavage. Cysteine-containing sequences were subjected to cleavage using a cocktail comprising 94% TFA, 2.5% H_2_O, 2.5% EDT, and 1% TIS. The resulting oligomers were precipitated in a cooled precipitation solution composed of MTBE and n-hexane (1:3). After centrifugation at 4000 rpm for 15 min, the resulting products were dried at RT under N_2_ gas flow. Size exclusion chromatography was employed to further purify the obtained oligomer solution utilizing an Äkta purifier system (GE Healthcare, Uppsala, Sweden. A solvent of 10 mM HCl/acetonitrile (ACN) in 7:3 used. The purified OAA solutions were subsequently lyophilized.

### 2.3. DBCO–PEG–Ligand Conjugate Synthesis

To synthesize DBCO–PEG_24_–L57 (DBCO–C–PEG_24_–TWPKHFDKHTFYSILKLGKH–COOH), we initiated the synthesis on 2-chlorotrityl resin preloaded with Fmoc protected His(Trt)–OH. The targeting peptide sequence was completed via automated SPSS. Subsequently, Fmoc–PEG_24_–OH and Fmoc–L–Cys(Trt)–OH were coupled to the sequence manually. The structure was cleaved from the resin by incubation with TFA:H_2_O:EDT:TIS (94:2.5:2.5:1) for 90 min. The resulting product was precipitated in 40 mL of a precooled mixture (1 part MTBE to 3 parts n-hexane). After centrifugation (4000 rpm, 4 °C, 15 min) the precipitate was dried and subsequently dissolved in 20 mM HEPES buffer. The pH of the solution was adjusted to 6.5, ensuring an adequate pH value for the conjugation of the sulfhydryl group, forming a thioether bridge with maleimide-DBCO. An excess of 2 eq of DBCO–maleimide dissolved in DMF was used. After incubating the mixture for 16 h at 4 °C, the resulting product was purified through preparative high-performance liquid chromatography (LaPrep system, VWR International GmbH, Darmstadt, Germany) using a SymmetryPrep C18 column (7 μm, 19 × 150 mm). The purified ligand was subsequent lyophilized. The scrambled DBCO–PEG peptide conjugates of L57, its retro-enantio version, and reL57–C terminated with cysteine were synthesized accordingly. The molecular structure of the ligands was further identified by the matrix-assisted laser desorption/ionization time-of-flight mass spectrometry.

### 2.4. RCD Synthesis

1,3-Dihydroxynaphthalene (100 mg) and KIO_4_ (400 mg) were dissolved in 100 mL ethanol. The resulting solution was transferred to a 200 mL poly(tetrafluoroethylene)-lined autoclave and heated at 180 °C for 2 h. After that, the autoclave was cooled naturally to RT. The solution was purified using silica column chromatography with an eluent mixture of methyl alcohol and dichloromethane (1:20). Subsequently, the RCD powder was obtained by freeze-drying.

### 2.5. Characterization of RCD

A transmission electron microscope (TEM) with 200 kV acceleration voltage (JEM-2100F, Tokyo, Japan) was used to investigate the morphologies of RCDs. The Fourier-transform infrared (FT-IR) spectrum was obtained from the Thermo Nicolet AVATAR 360 FT-IR using the KBr pellet method. A ^1^H NMR was performed on a DMX 500 (Bruker, Bremen, Germany) with deuterated chloroform as the solvent. The ultraviolet-visible absorption spectra were recorded on a UV-2401PC absorption spectrometer (Shimadzu, Melbourne, Australia). The fluorescence spectra were measured on the FS5 fluorescence spectrometer (Edinburgh, UK). The RCDs were dispersed in water or PBS with various pH and different UV exposure (ZW14S15W) periods for photostability measurements at RT. Optical and photographs of RCDs were taken by a common Canon camera under daylight and UV light (395 nm).

### 2.6. RCD@OAA Preparation

First, RCD and OAA HEPES buffers with the same volumes were prepared, then the RCD@OAA were formed by mixing OAA and RCD solution by pipetting (10 times), followed by incubation at 37 °C for 1 h. A dose titration of RCD@1696 was performed by adding different amounts of oligomer to 1 µg RCD.

### 2.7. RCD@1696–Ligands and RCD@1696–PEG Preparation and Characterization

RCD@1696–ligands and controls were prepared based on the click chemistry between the azide group of ***1696*** and the DBCO group of the ligands or the PEG control. The amount of DBCO agents required was calculated according to the molar ratios between the azide groups to DBCO groups. In detail, RCDs@OAA were dissolved in HEPES buffer (20 mM, pH 7.4), and simultaneously, DBCO–PEG–ligand or DBCO–PEG conjugates HEPES buffer with different molar ratio (0.5, 2, and 4) were also prepared. The final formulations preparation was performed by quickly mixing the two solutions using a pipette and subsequently incubating at RT for 4 h.

The obtained OAAs were confirmed by ^1^H NMR spectroscopy at 400 MHz (JEOL, Tokyo, Japan). Deuterium oxide at 4.79 ppm was used as the internal standard to correct the chemical shifts. Imidazole peaks were taken to normalize the peak. Spectra were analyzed using MestreNova (Mestrelab Research, Santiago de Compostela, Spain). The hydrodynamic size and zeta potential of formulations were tested by a Zetasizer Nano ZS (Malvern, Worcestershire, UK).

### 2.8. Ellman’s Assay

A 170 µL working solution containing 2.44 mL Ellman buffer (1 mM ethylenediaminetetraacetic acid, 0.2 M Na_2_HPO_4_, pH 8.0), and 60 µL of reagent (5,5′-dithiobis-(2-nitrobenzoic acid) solution) were mixed with 30 µL of RCD@1696 solution. The tubes were incubated at 37 °C under the exclusion of light for 15 min, and the absorption at 412 nm was recorded using a spectrophotometer (Thermo Scientific). The calculation of free thiol percentage was determined by referencing the theoretical amount of thiols, which was 100%, assuming complete cleavage.

### 2.9. TCEP Assay

TCEP was applied as a reducing agent to cleave the disulfide bridge between cysteines. Then, 0.5 M TCEP in sterile RNase-free water was mixed with 100 µL RCD@1696 HEPES solution to obtain a final TCEP concentration of 0.005 M. After incubating at 37 °C or 4 °C for different time periods, the relative hydrodynamic size and zeta potential of the formulations were recorded.

### 2.10. Cell Viability Assay

One day before the treatments, human glioblastoma-astrocytoma U87MG cells were seeded on 96-well plates with a density of 5 × 10^3^ cells/well. After 24 h, the cells were incubated with RCD, ***1696***, RCD@1696, or RCD@1696–ligand for another 24 h. Then, a standard MTT assay was performed to test the relative cell viability.

### 2.11. Cellular Internalization

U87MG cells and human umbilical vein endothelial cells (HUVECs) were plated on 96-well plates at a density of 8 × 10^3^ cells per well, respectively. Following incubation overnight, different formulations were added to the cell medium. After incubation for different time periods, the cells were washed with PBS and 500 I.U. heparin to remove the particles attached to the cell surface. The cells were finally suspended in PBS containing 20% FBS and analyzed using flow cytometry in the ECD channel (Beckman Coulter, Fullerton, CA, USA). The data were analyzed by the FlowJo 7.6.5 software (FlowJo, Ashland, CA, USA).

### 2.12. Ligand Competition Assay

hCMEC/D3 cells (2 × 10^4^ per well) were seeded on 96-well plates in 100 μL of medium and incubated for 24 h. Next, the cells were treated with scr-L57 (5 µM), receptor-associated protein (RAP, 5 µM), and L57 (5 µM), respectively. The cells were incubated with the respective compounds for 45 min on ice. Afterwards, 6-carboxyfluorescein (6-FAM)-labeled reL57 was added to the medium and incubated for an additional 20 min at 37 °C. Following this incubation, the cells were washed with 1 × PBS, suspended in PBS buffer containing 20% FBS, and analyzed using flow cytometry.

### 2.13. Statistical Analysis

Data were expressed as the means ± standard deviation (SD) of at least three independent experiments. The statistical significance of the experiments was determined using the two-tailed Student’s *t*-test (**** *p* ≤ 0.0001, *** *p* ≤ 0.001, ** *p* ≤ 0.01, * *p* ≤ 0.05; n.s., not significant).

## 3. Results

### 3.1. Design and Synthesis of Four-Armed OAA for Caging of RCD

**Figure 1:**
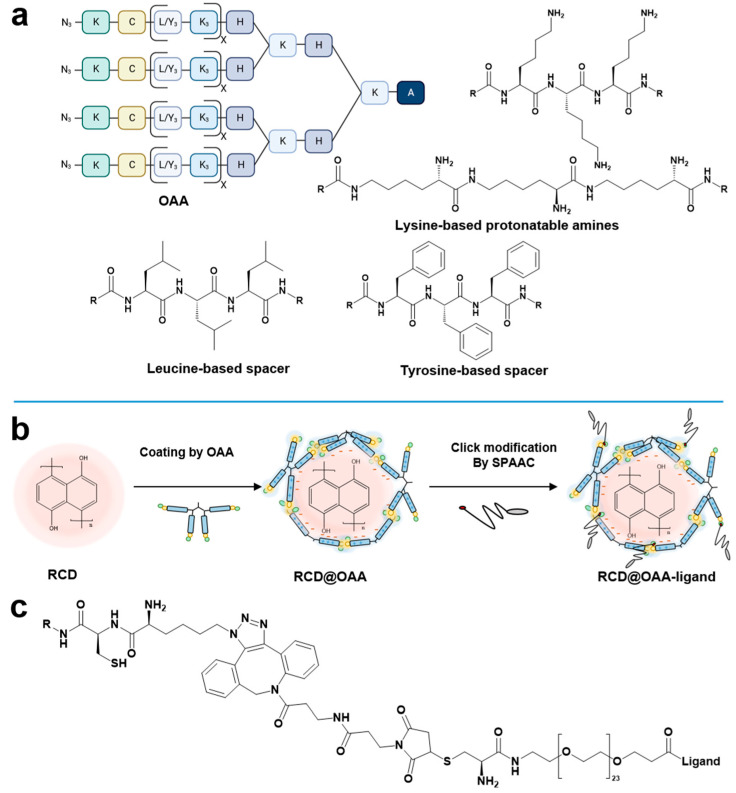
Schematic illustration of sequence-defined oligomers and click chemistry-supported RCD delivery system. (**a**) Chemical structures of four-armed oligomers with N-terminal azidolysine for post-functionalization, cysteine for forming bioreversible disulfide bridges, tyrosine and leucine tripeptides, respectively, adopted as aromatic as well as aliphatic spacers, α-amino or ε-amino-linked lysine tripeptides as cationic components defining the length of each arm. A: alanine; K: lysine; H: histidine; L: leucine; Y: tyrosine; C: cysteine; KN_3_: azidolysine; x: number of repetitions. Detailed sequences are listed in Table 1. (**b**) Schematic illustration of preparation of RCD@OAA-ligand. (**c**) Chemical structure of four-armed oligomer linked with ligands via copper-free SPAAC modification.

For coating and caging RCDs (termed ‘RCD@OAA’), sequence-defined four-armed OAAs were fabricated via SPSS methodology analogously as previously reported [36,37]. The design of these OAAs was based on the following rationales (Figure 1a):

(i) Each arm of the OAAs contained two or three lysine tripeptides with corresponding numbers of positively charged amines, facilitating successful layer coating of the negatively charged hydroxynaphthalene-derived RCDs via the electrostatic interactions. Preferably, the lysine units were incorporated as ε-amino amidated lysine instead of the standard α-amino amidated lysine residues, increasing the contour length of each arm without changing the number of amino acids and positively charged amines, respectively.
(ii) To enhance the stability of RCD@OAA, alternating with two lysine tripeptide blocks, two blocks of tyrosine or leucine tripeptides were included, serving either as aromatic or hydrophobic interaction domains stabilizing the coating of RCDs.
(iii) Thiol groups of cysteines located close to the four N-terminal ends were included that should cage the coated RCDs by disulfide bridge formation.
(iv) To enable controlled shielding and targeting of caged RCDs with ligand–PEG–DBCO via SPAAC, N-terminal azidolysines were included.

Five four-armed structures (oligomer ***1658***, ***1664***, ***1696***, ***1768***, and ***1769***) were selected for further investigation (see Table 1, and Supplementary Materials Figures S1–S5) after a preliminary library screen of 21 oligomers [38]. To unravel the impact of the four-armed OAAs’ structure on the caging of RCDs, it was crucial to analyze each factor individually. Specifically, to clarify the effect of arm length, we adopted ε-amino linked lysines as an alternative to the standard α–amino linked lysines, increasing the contour length of each arm without changing the sequence and the corresponding number of positively charged primary amines (oligomer ***1664*** versus ***1696***, ***1768*** versus ***1769***). Two different alternating tripeptide spacer units were introduced, to explore π–π stacking interaction between oligomers and hydroxynaphthaline-derived RCDs. These tripeptides are either composed of tyrosines as aromatic spacers or of leucines as aliphatic hydrophobic spacers (oligomer ***1658*** versus ***1664***). A similar comparison was also utilized between oligomer ***1696*** and its counterpart ***1796***, with the latter lacking tyrosine tripeptide motifs.

### 3.2. LRP1 Targeted DBCO–PEG–Ligand Peptide Conjugates

For PEG-shielding and LRP1 targeting of caged RCD@OAA nanoparticles (Figure 1b), a series of DBCO–PEG–ligand conjugates were synthesized (Figure 1c and Table 2). In particular, the novel retro-enantio LRP1 ligand (reL57) containing reversed N- to C-terminal order of amino acids (sequence: DBCO–CPEG_24_–hkglklisyfthkdfhkpwt–COOH; small single letter codes represent (D) amino acids) was attached to a monodisperse PEG-spacer (24 monomer units) and DBCO for subsequent click reaction (see also Figure S6, Supplementary Materials). The strategy of retro-enantio peptide ligands was previously reported as an encouraging strategy for developing protease-resistant ligands [39,40,41,42]. Conjugates of the standard peptide L57 sequence, of scrambled L57 sequence, and reL57 containing a terminal cysteine were synthesized as controls. As shown in Figures S6–S9 in the Supplementary Materials, the successful synthesis of these ligands was accurately demonstrated by matrix-assisted laser desorption/ionization time-of-flight mass spectrometry spectra. Except for reL57-C, the N-terminus of the other three ligands was linked to the DBCO group and a monodisperse PEG_24_ spacer to facilitate modification.

### 3.3. RCD Synthesis and Characterization

**Figure 2:**
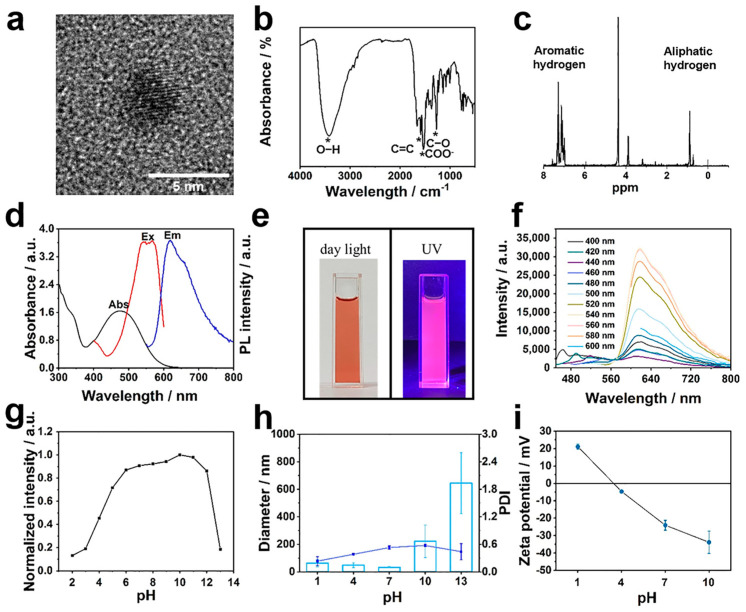
Characterization of RCD. (**a**) TEM image, (**b**) FT-IR absorption spectrum, and (**c**) ^1^H NMR spectrum of RCD. * represents the peak corresponding to the chemical bond. (**d**) UV–vis absorption and fluorescence spectra of RCD aqueous solution. The black, red, and blue lines represent UV absorption, excitation (Ex), and emission (Em) fluorescence spectra, respectively. (**e**) Solution color photograph of RCD in deionized water under daylight (**left**) and UV light (**right**). (**f**) Fluorescence emission intensity of RCD aqueous solution at different excitation wavelengths. (**g**) Normalized fluorescence emission intensity, (**h**) hydrodynamic particle size (number; light blue column), PDI (dark blue line), and (**i**) zeta potential of RCD solution under series pH conditions. Data are presented as mean ± SD (n = 3).

The fluorescent RCDs were fabricated using a solvothermal condensation carbonization and dehydrogenative planarization strategy, as described in a previous paper [13]. Tunable near-infrared emission was obtained by introducing large π-conjugated structures derived from 1,3-dihydroxynaphthalene. A TEM measurement revealed that the RCDs possessed a uniform size and were narrowly distributed around 3.5 nm in diameter, which was smaller than the permeable size of the glomerular basement membrane (Figure 2a). FT-IR spectroscopy confirmed the presence of C=O at 1536 cm^−1^ and C–O at 1271 cm^−1^ corresponding to the stretching vibration of the carboxyl group, and the peak at 1588 cm^−1^ corresponding to the π-conjugated system could also be observed (Figure 2b). Moreover, ^1^H NMR was also utilized to confirm the presence of aromatic H signals in the range of 7.0–8.0 ppm and aliphatic H signals (Figure 2c). Together with the abovementioned results, it is strongly suggested that the synthesized RCD owned sp^2^-hybridized skeleton and reactive carboxyl groups, allowing electrostatic as well as aromatic interactions for further four-armed oligomers coating and implying relatively strong red emission.

Precursors’ structures and reaction conditions underlie the optical properties of RCDs; as shown in Figure 2d, the ultraviolet-visible spectrum of RCDs revealed a distinct absorption peak centered at 476 nm, indicating the formation of conjugated sp^2^-hybridized carbon cores, while weak peaks at 300–350 nm corresponded to the n-π* transitions of RCDs. The as-prepared RCDs could be well dispersed in aqueous media and resulted in a red solution under daylight, which turned into bright pink fluorescence under UV light irradiation (Figure 2e). Significantly, the fluorescence emission intensity of RCDs depended on the excitation wavelength, with the highest photoluminescence emission observed at 618 nm when excited with a wavelength of 540 nm, resulting in red fluorescence (Figure 2d,f). Previous papers have demonstrated that fluorescence probes can exhibit pH- and concentration-dependent switchable fluorescent properties. Figure 2g and Figure S10 in the Supplementary Materials illustrate the pH-triggered fluorescent quenching phenomenon of RCDs, which was observed under both acidic and alkaline conditions. Notably, the related intensity dropped dramatically to 0.2 when the pH was adjusted from 12 to 13, and gradually decreased in acidic aqueous solutions. It is worth noting that changing the pH significantly altered the dispersion and solubility of RCDs in aqueous media. At normal physiological pH (6.8–7.4), the average hydrodynamic diameter of RCDs, as measured by dynamic light scattering, was 33 nm, but the polydispersity index (PDI) was unfavorable for biological application (Figure 2h). Moreover, similar to other conventional fluorescent probes [43], an aggregation-induced quenching effect was also proved in high concentration-RCD systems (Figure S11, Supplementary Materials). In contrast to small organic molecules, the synthesized carbon nanodots hold outstanding photostability to resist photobleaching, which is another critical feature of medical imaging (Figure S12, Supplementary Materials).

### 3.4. Screening of Four-Armed OAAs for RCD Coating

**Figure 3:**
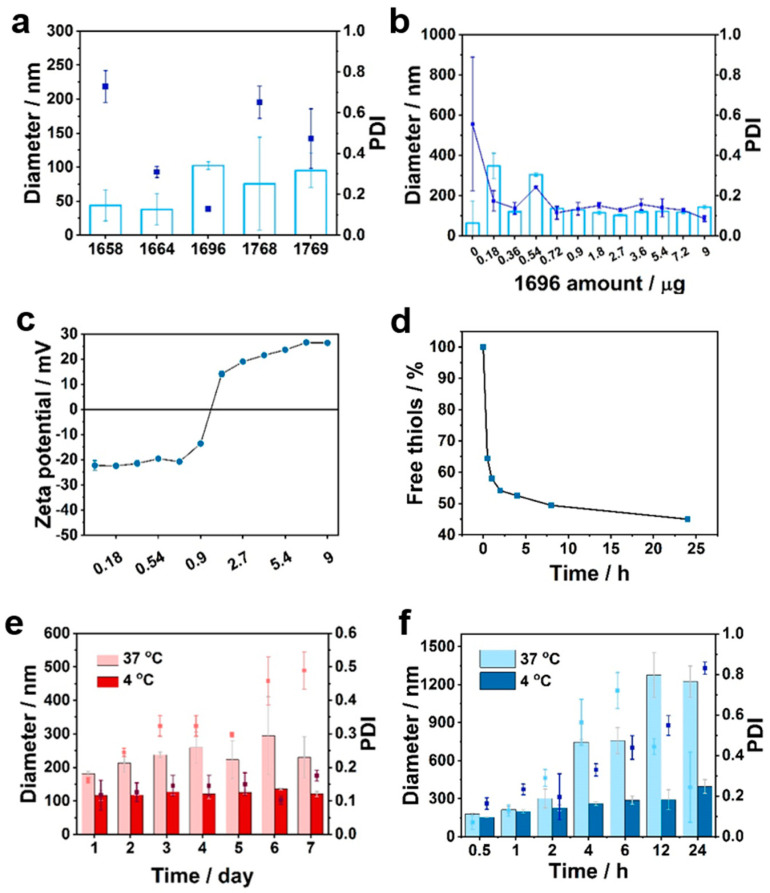
Screening of four-armed oligomers for RCD. (**a**) Particle diameter (number) of RCD@OAA formulations (***1658***, ***1664***, ***1696***, ***1768***, and ***1769***). (**b**,**c**) Dose titration of RCD@1696 containing 0 to 9 µg oligomer ***1696*** and 1 µg RCD. (**d**) Ellman’s assay for RCD@1696 conjugation with time dependency of incubation. Stability analysis of RCD@1696 upon incubation at 37 °C or 4 °C (**e**) without or (**f**) with 0.05 M TCEP added. For figures involving particle diameter and PDI, the columns represent size, the dots represent PDI. Data are presented as mean ± SD (n = 3).

The negative zeta potential of RCDs (around –30 mV) promoted the development of sequence defined, positively charged, four-armed OAAs for addressing aggregation and stability issues. Successful synthesis was confirmed by ^1^H NMR spectra (Figure 2i and Figure S1–S5, Supplementary Materials). According to the principles mentioned above, cationic carriers and RCDs spontaneously assembled in water, generating RCD@OAA formulations with positive zeta potentials ranging from +15 to +25 mV (Figure S13, Supplementary Materials). However, particle sizes and PDI varied widely, indicating that only specific OAA sequences were suited to cage and entrap the RCDs (Figure 3a). In detail, to comprehensively evaluate the structure-activity relationship between RCD and OAA, it was crucial to compare different attributes one by one. For instance, comparing the oligomers ***1664***, ***1696***, ***1768***, and ***1769*** with each other; oligomer ***1768,*** retaining an arm length of ~4.32 nm, was the shortest among the four, and resulted in particles with a PDI of 0.7, and therefore did not fulfill caging and entrapment requirements. Conversely, oligomer ***1769*** owns an arm length of ~8.85 nm, which is attributed to its ε-lysine linked configuration, as opposed to the α-lysine configuration of ***1768***. Encouragingly, RCD@OAA based on ***1769*** possessed a size of 95 nm and PDI of 0.4; it was more efficient than its counterpart ***1768*** because of the increased length. A similar result was observed for ***1664*** (length of ~5.40 nm) and its counterpart ***1696*** (length of ~8.42nm), with the latter being more effective due to ε-amino-linked lysine configuration, compared to the α-amide-linked configuration of ***1664***.

Furthermore, the presence of aromatic groups is another considerable factor in RCD@OAA formulation. To investigate this aspect, we incorporated leucines as an aliphatic amino acid block and tyrosines as an aromatic amino acid block within the oligomers backbone, respectively. Oligomer ***1658*** was constructed with leucine units; its particle showed a diameter of approximately 44 nm and a PDI of 0.7, while its tyrosine counterpart, oligomer ***1664***, resulted in particles with a lower PDI. The significance of incorporating tyrosine units into the sequence was further proven when comparing oligomers ***1769*** and ***1696***. The arms of ***1769*** consist solely of lysine units and are terminated with an azidolysine, whereas ***1696*** includes an additional tyrosine triblock. The difference between the resulting particles in size and PDI was immense. Given the success of ***1696***, it can be concluded that both the effects of length increase and tyrosine π-electron stacking play decisive roles in the 3D entrapment of RCD by four-armed OAA.

To further analyze the relationship between RCDs and best-performing OAAs, a dose titration (*w/w*) was carried out. Notably, after incorporating oligomer into the system, the PDI of RCD@1696 decreased with raising amounts of ***1696*** (Figure 3b). The shift point for the zeta potential of the formulations was observed within the range of 0.9–1.8 μg of ***1696***, indicating carrier saturation in the formulation (Figure 3c). The optimal incubation time was confirmed, the coating of the formulation happened almost instantly, within 0.5 h (Figure S14, Supplementary Materials). The crosslinking reaction of the thiol groups within the cysteines, which leads to a covalent disulfide bond, required more time and was introduced as another mechanism for RCD entrapment. An Ellman’s assay was adopted to quantify the amount of free thiol groups in the formulation (Figure 3d) [44,45]. The amount of free thiol groups in RCD@1696 dramatically decreased to 65% after 0.5 h incubation and below 53% within 4 h. In addition, the bioreversible disulfide bridge, together with the aromatic interaction of tyrosines and amide hydrogen bonds, endowed RCD@1696 with excellent storage stability, which was confirmed by testing the change in size distribution at 4 °C (Figure 3e). After 4 days of incubation at 37 °C, the corresponding PDI raised to 0.4, which was still acceptable for further biological application. Additionally, TCEP, a typical reducing agent, was applied to cleave the disulfide bridge between cysteine. As expected, the size and PDI for RCD@1696 under TCEP-reduced conditions exhibited an evident increase compared to the untreated conditions (Figure 3f).

### 3.5. Click Modification of RCD@1696 with DBCO–PEG–Ligand Conjugates

**Figure 4:**
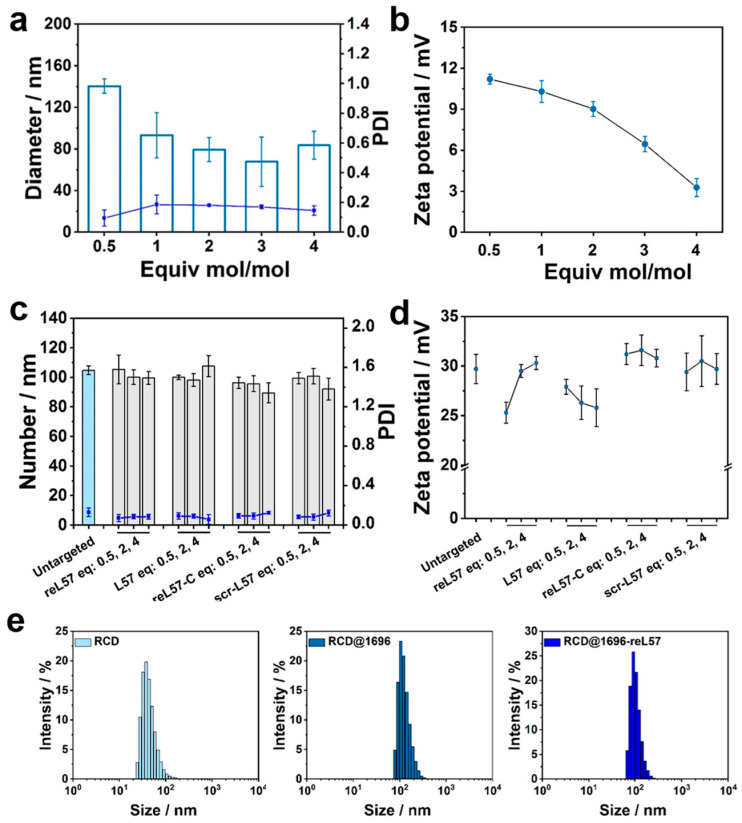
Characterization of RCD@1696 DBCO–PEG or DBCO–PEG–ligand conjugates. (**a**) Size distribution (number), PDI, and (**b**) zeta potential of RCD@1696-modified with DBCO–PEG5000 with different molar eq. (**c**) Size distribution (number), PDI, and (**d**) zeta potential of RCD@1696-modified with DBCO–reL57 and controls with different molar eq. (**e**) Intensity size distribution of three kinds of RCD formulations. For figures involving particle diameter and PDI, the columns represent size, the dots represent PDI. Data are presented as mean ± SD (n = 3).

Encouraged by the superior performance of ***1696*** in enhancing colloidal stability and aqueous dispersibility, the oligomer was selected for subsequent modification with shielding and targeting domains. The obtained formulation was subsequently conjugated with different equivalents of DBCO–PEG5000 using click chemistry to generate PEG-modified nanocarriers (mol/mol) (Table 2). The effect of the PEG feed ratio on the physiochemical properties of RCD@1696 was revealed. The modified nanocarriers displayed homogeneous particle formation, with a PDI less than 0.2; surprisingly, the size of RCD@1696 slightly decreased to 84 nm after modification with 4 eq of DBCO–PEG5000 (Figure 4a). The related zeta potential gradually decreased from +11 to +3 mV with an increasing PEG-modified ratio, which indicates the shielding effect of PEG and the successful SPAAC (Figure 4b).

Most importantly, the presence of azido functionality on the surface RCD@1696 was a prerequisite for subsequent ligand clicking. Herein, four distinct types of LRP1 ligand peptides and control domains were designed and synthesized according to SPPS, and subsequently coupled with monodisperse Fmoc–PEG_24_–OH. To avoid degradation and achieve effective LRP1 targeting, the protease-resistant retro-enantio peptide reL57 was developed. As shown in Table 2, in contrast to *L*-configuration peptides in organisms, reL57 contains all amino acids in *D*-configuration and reverse N to C-terminal order (sequence: DBCO–C–PEG_24_–hkglklisyfthkdfhkpwt-COOH). A conjugate containing the scrambled sequence DBCO–C–PEG_24_–KPFKHGTDLLKHFWYSHTKI–COOH, denoted as scr-L57, served as a control reagent. Additionally, the PEG_24_ shielding domain (including 24 ethylene oxide monomer units) and reL57-C terminated with cysteine instead of DBCO were employed as negative controls. As shown in Figure 4c, all ligand conjugations displayed excellent size distribution in the range of 90–110 nm. There was no significant difference after modification with 0.5 to 4 eq of DBCO ligand, and a higher ratio with more than one equivalent did not induce any aggregation (PDI < 0.2), illustrating that the ligand feed ratio displayed a negligible influence on the physiochemical properties of RCD@1696. Minimal changes in the zeta potential of the dispersion could be observed after modification with reL57 and its controls (Figure 4d). Not surprisingly, the hydrodynamic diameter of RCD (approximately 33 nm) was smaller than that of RCD@1696, whether with or without 0.5 eq of reL57 modification (Figure 4e).

### 3.6. Evaluation of reL57 as Targeting Ligand for Receptor-Mediated RCD Delivery

**Figure 5:**
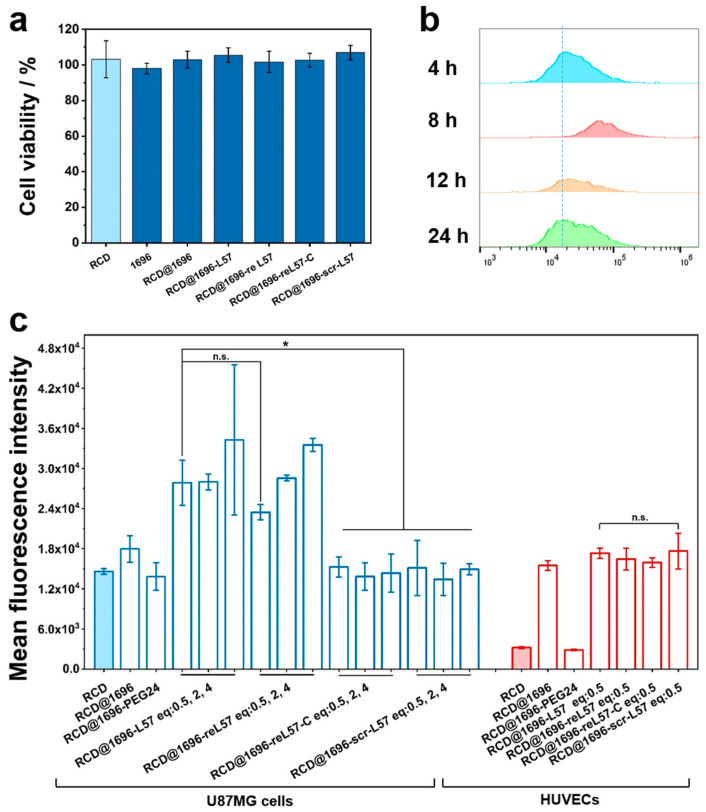
Characterization of reL57 and control-ligands-based RCD@1696 delivery system. (**a**) Cell viability of U87MG cells treated with RCD, oligomer ***1696***, RCD@1696, or RCD@1696–ligands for 24 h. Data are presented as mean ± SD (n = 5). (**b**) Cellular uptake assay of naked RCD (25 µg mL^−1^) in U87MG cells measured by flow cytometry after 4 h, 8 h, 12 h, and 24 h incubation. (**c**) Quantitative analysis of RCD fluorescence intensity of RCD-related ligands system. The U87MG cells and HUVECs were incubated with formulations with different molar ratios for 8 h. The initial concentrations of carbon nanodots for naked RCD and RCD@1696, or RCD@1696–ligands, were equal, and the amount of oligomer for different formulations was also the same. Data are presented as mean ± SD (n = 3). Statistical significances were calculated by Student’s *t*-test. * *p* ≤ 0.05. n.s., not significant.

Before confirming the feasibility of receptor-mediated delivery in vitro, the cellular cytotoxicity of RCD with and without oligomer, ligand–PEG, and PEG-modification was evaluated in human glioblastoma U87MG cells, which express LRP1 at a high level [46]. After co-culturing U87MG cells with RCDs, oligomer ***1696***, RCD@1696, and RCD@1696–ligands for 24 h, respectively, the superior biosafety of the obtained receptor-mediated delivery system was validated in a cell viability assay as depicted in Figure 5a. Encouraged by the negligible cytotoxicity, we further explored the endocytosis of the LRP1-derived peptide system, intending to enhance RCD delivery. Different incubation times (4 h, 8 h, 12 h, and 24 h) of RCDs with U87MG cells were quantified by flow cytometry in the ECD channel (Ex: 561 nm, Em: 590–630 nm), which was closest to the RCD emission and excitation wavelengths. Notably, after 8 h of incubation, the cellular uptake of RCDs was at its highest level, with the mean fluorescence intensity approximately one-fold higher than in the case of 4 h incubation (Figure 5b and Figure S15, Supplementary Materials). The same tendency was visible at lower RCD concentrations.

Encouraged by this result, to elucidate the impact of ligand molar ratio on endocytosis, the cellular uptake of the RCD-related ligand system was analyzed (Figure 5c). Consistent with our expectations, L57 and reL57–PEG-modified nanoparticles mediated the highest levels of cellular uptake at different equivalents in LRP1 highly expressing cells, while nanoprobes modified with control ligands (RCD@1696–reL57–C as well as RCD@1696–scrL57) and naked nanoparticles exhibited the lowest uptake. Moreover, RCD@1696–reL57 formulations also demonstrated a ligand molar ratio-dependent uptake characteristic. In particular, RCDs alone generated a signal intensity of around 15,000, whereas the intensity signal of the corresponding conjugate RCD@1696 slightly increased to 18,000 due to electrostatic adsorption. It has been well investigated that in the case of non-targeted nanoprobes, cellular uptake mainly relies on physicochemical properties, including surface charge, size, shape, and so on [47,48]. Thus, the spherical cationic carbon nanodots modified with four-armed oligomer enabled the system to easily attach to negatively charged lipid bilayers, inducing improved predominant internalization via clathrin-mediated endocytosis [49]. By comparison, covalent conjugation of PEG_24_–DBCO and RCD@1696 resulted in a slight decrease of the intensity, indicating reduced uptake due to shielding effect.

**Figure 6:**
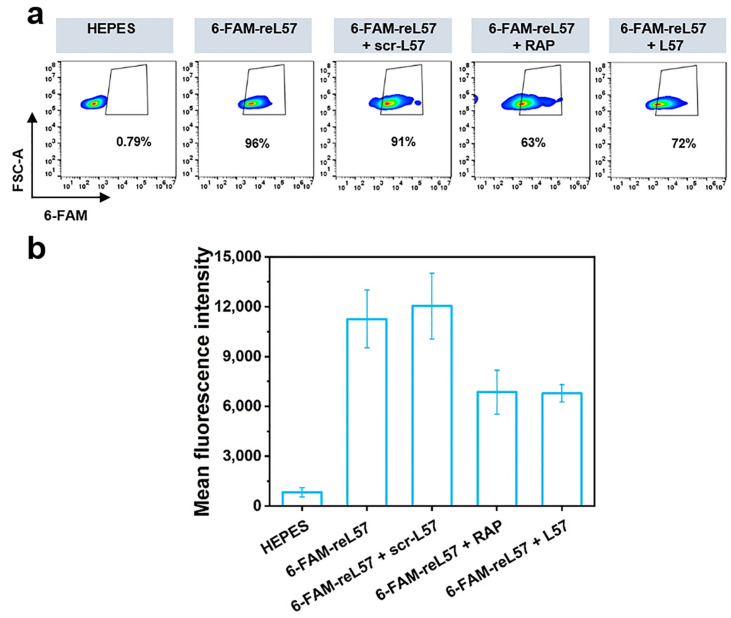
Ligand competition assay to confirm reL57 target site. (**a**) Competitive inhibition of 6-FAM-reL57 binding to hCMEC/D3 cells in the presence of scr-L57 (5 µM), RAP (5 µM), and L57 (2.5 µM), respectively. HEPES was used as the negative control. reL57 without any treatment was adopted as the positive control. (**b**) Corresponding quantification of 6-FAM fluorescence intensity. Data are presented as mean ± SD (n = 3).

To verify that the uptake enhancement corresponded to LRP1 expression, we further assessed the related endocytosis in LRP1-low-expressing HUVECs [25]. Compared with naked RCD, incubation of the cells with RCD@1696 for 8 h induced a 4.8-fold improvement in uptake. Consistent with the conclusion found in U87MG cells, modification with PEG_24_ significantly decreased the cellular uptake, proving efficient PEG shielding. In contrast, there was no distinct difference between the four ligand-modified groups. This suggests that the enhanced delivery is based on the LRP1 receptor-mediated endocytosis. To further validate the specific interaction between reL57 and LRP1, we employed the receptor-associated protein (RAP), a well-known inhibitor that hinders ligand binding to LRP1 [50,51]. In detail, RAP, scr-L57, and L57 were added to the cells and incubated for 45 min at 0 °C before addition of reL57, respectively. As shown in Figure 6a,b, compared with the control group (6-FAM-reL57 + scr-L57), quantification of 6-FAM fluorescence intensity in hCMEC/D3 cells proved an obvious blocking in the RAP-treated cells with 43% fluorescence decrease. Similarly, L57 successfully inhibited reL57 binding.

## 4. Conclusions

We successfully designed and synthesized new lysine (ε-amino)-linked cationic four-armed oligomers for coating and caging dihydroxynaphthalene-derived RCDs and a targeting retro-enantio peptide ligands for delivery to LRP1 overexpressing cells. The leading cationic four-armed oligomer ***1696***, spanning a contour length of ~8.42 nm for each arm, stood out for containing two ε-amino-linked lysine tripeptide units alternating with two aromatic tyrosine tripeptide units, demonstrating superior modification due to a combination of electrostatic charge interactions and aromatic π-electron stacking bonds. The increased stability achieved by cysteine-based disulfide bridge bonding was indirectly proved by subsequent destabilization upon adding the reducing agent TCEP. The resulting caged RCD@1696 exposed N-terminal reactive azido units which could be click-modified with DBCO–PEG-ligands; in comparison to other PEG or ligands controls, the DBCO–PEG-modified peptide reL57-linked RCD@1696 achieved efficient targeting and delivery to LRP1 highly-expressing U87MG cells.

## Figures and Tables

**Table 1:** Sequences of four-armed OAA. Sequences (left to right) from N- to C-terminus.

OAA ID Number	Sequence # (N → C)	Length of Arms *
***1658***	[[H_2_N-(N_3_)K-C-L_3_-K_3_-L_3_-K_3_-H]_2_-α,εK-H]_2_-α,εK-A-COOH	5.40 nm
***1664***	[[H_2_N-(N_3_)K-C-Y_3_-K_3_-Y_3_-K_3_-H]_2_-α,εK-H]_2_-α,εK-A-COOH	5.40 nm
***1696***	[[H_2_N-(N_3_)K-C-Y_3_-εK_3_-Y_3_-εK_3_-H]_2_-α,εK-H]_2_-α,εK-A-COOH	8.42 nm
***1768***	[[H_2_N-(N_3_)K-C-K_9_-H]_2_-α,εK-H]_2_-α,εK-A-COOH	4.32 nm
***1769***	[[H_2_N-(N_3_)K-C-εK_9_-H]_2_-α,εK-H]_2_-α,εK-A-COOH	8.85 nm

# Unless indicated differently, amino acids were linked by standard α–amino amide bonds. For ***1696*** and ***1769***, ε-amino-linked lysine tripeptides (εK) were incorporated. * Contour length of the four arms spanning from terminal azidolysine to the first histidine before 4-fold branching.

**Table 2:** Sequences of the DBCO–PEG–ligand, control ligand containing terminal cysteine, and DBCO–PEG reagents synthesized via SPPS. Sequences (left to right) from N- to C-terminus. Small single letter codes: (*D*) amino acids.

Peptide Name	Sequence (N → C)
reL57	DBCO–C–PEG_24_-hkglklisyfthkdfhkpwt-COOH
L57	DBCO–C–PEG_24_-TWPKHFDKHTFYSILKLGKH-COOH
scr-L57	DBCO–C–PEG_24_-KPFKHGTDLLKHFWYSHTKI-COOH
reL57-C	H_2_N–C–hkglklisyfthkdfhkpwt-COOH
PEG24 shielding domain	DBCO–PEG_24_-COOH
PEG5000 shielding domain	DBCO–PEG 5 kDa

## Data Availability

The data supporting the findings of this study are accessible within the article and its Supplementary Materials.

## Supplementary Materials

The following Supplementary Materials can be downloaded at: https://www.mdpi.com/article/10.3390/polym15204039/s1, Table S1: Source of protected amino acids used in peptide synthesis; Figure S1: Molecular structure and ^1^H NMR spectrum of ***1658*** four-armed structure, recorded in D_2_O; Figure S2: Molecular structure and ^1^H NMR spectrum of ***1664*** four-arm structure, recorded in D_2_O; Figure S3: Molecular structure and ^1^H NMR spectrum of ***1696*** four-arm structure, recorded in D_2_O; Figure S4: Molecular structure and ^1^H NMR spectrum of ***1768*** four-arm structure, recorded in D_2_O; Figure S5: Molecular structure and ^1^H NMR spectrum of ***1769*** four-arm structure, recorded in D_2_O; Figure S6: Molecular structure and MALDI-TOF-MS spectrum of reL57; Figure S7: Molecular structure and MALDI-TOF-MS spectrum of L57; Figure S8: Molecular structure and MALDI-TOF-MS spectrum of scr-L57; Figure S9: Molecular structure and MALDI-TOF-MS spectrum of re-L57-C; Figure S10: Fluorescence emission intensity of RCD aqueous solution under series pH conditions; Figure S11: Fluorescence emission intensity of RCD aqueous solution with different concentrations; Figure S12: Fluorescence emission intensity and normalized fluorescence emission intensity of RCD aqueous solution after irradiation for different times; Figure S13: Zeta potential of RCD@OAA (***1658***, ***1664***, ***1696***, ***1768***, and ***1769***). Figure S14: Hydrodynamic particle size, PDI, and zeta potential of RCD@1696 solution after incubation for different times; Figure S15: Cellular uptake assay of naked RCD in U87MG cells measured by flow cytometry and the corresponding quantitative analysis of fluorescence intensity.

## References

[1] Ostrom Q.T., Price M., Neff C., Cioffi G., Waite K.A., Kruchko C., Barnholtz-Sloan J.S. (2022). CBTRUS Statistical Report: Primary Brain and Other Central Nervous System Tumors Diagnosed in the United States in 2015–2019. Neuro. Oncol..

[2] Xie R., Wu Z., Zeng F., Cai H., Wang D., Gu L., Zhu H., Lui S., Guo G., Song B. (2021). Retro-Enantio Isomer of Angiopep-2 Assists Nanoprobes across the Blood-Brain Barrier for Targeted Magnetic Resonance/Fluorescence Imaging of Glioblastoma. Signal Transduct. Target. Ther..

[3] Demchenko A.P. (2020). Photobleaching of Organic Fluorophores: Quantitative Characterization, Mechanisms, Protection. Methods Appl. Fluoresc..

[4] Helmerich D.A., Beliu G., Matikonda S.S., Schnermann M.J., Sauer M. (2021). Photoblueing of Organic Dyes Can Cause Artifacts in Super-Resolution Microscopy. Nat. Methods.

[5] Xia C., Zhu S., Feng T., Yang M., Yang B. (2019). Evolution and Synthesis of Carbon Dots: From Carbon Dots to Carbonized Polymer Dots. Adv. Sci..

[6] Kütahya C., Zhai Y., Li S., Liu S., Li J., Strehmel V., Chen Z., Strehmel B. (2021). Distinct Sustainable Carbon Nanodots Enable Free Radical Photopolymerization, Photo-ATRP and Photo-CuAAC Chemistry. Angew. Chem. Int. Ed..

[7] Rigodanza F., Burian M., Arcudi F., Đorđević L., Amenitsch H., Prato M. (2021). Snapshots into Carbon Dots Formation through a Combined Spectroscopic Approach. Nat. Commun..

[8] Lesani P., Mohamad Hadi A.H., Lu Z., Palomba S., New E.J., Zreiqat H. (2021). Design Principles and Biological Applications of Red-Emissive Two-Photon Carbon Dots. Commun. Mater..

[9] Lu S., Sui L., Liu J., Zhu S., Chen A., Jin M., Yang B. (2017). Near-Infrared Photoluminescent Polymer–Carbon Nanodots with Two-Photon Fluorescence. Adv. Mater..

[10] Li D., Han D., Qu S.-N., Liu L., Jing P.-T., Zhou D., Ji W.-Y., Wang X.-Y., Zhang T.-F., Shen D.-Z. (2016). Supra-(Carbon Nanodots) with a Strong Visible to Near-Infrared Absorption Band and Efficient Photothermal Conversion. Light Sci. Appl..

[11] Guo X.-L., Ding Z.-Y., Deng S.-M., Wen C.-C., Shen X.-C., Jiang B.-P., Liang H. (2018). A Novel Strategy of Transition-Metal Doping to Engineer Absorption of Carbon Dots for Near-Infrared Photothermal/Photodynamic Therapies. Carbon.

[12] Zhang T., Cheng Q., Lei J.H., Wang B., Chang Y., Liu Y., Xing G., Deng C., Tang Z., Qu S. (2023). Constructing Oxygen-Related Defects in Carbon Nanodots with Janus Optical Properties: Noninvasive NIR Fluorescent Imaging and Effective Photocatalytic Therapy. Adv. Mater..

[13] Wang Z., Yuan F., Li X., Li Y., Zhong H., Fan L., Yang S. (2017). 53% Efficient Red Emissive Carbon Quantum Dots for High Color Rendering and Stable Warm White-Light-Emitting Diodes. Adv. Mater..

[14] Zhao S., Yan L., Cao M., Huang L., Yang K., Wu S., Lan M., Niu G., Zhang W. (2021). Near-Infrared Light-Triggered Lysosome-Targetable Carbon Dots for Photothermal Therapy of Cancer. ACS Appl. Mater. Interfaces.

[15] Han Y., Liu H., Fan M., Gao S., Fan D., Wang Z., Chang J., Zhang J., Ge K. (2022). Near-Infrared-II Photothermal Ultra-Small Carbon Dots Promoting Anticancer Efficiency by Enhancing Tumor Penetration. J. Colloid Interface Sci..

[16] Arvanitis C.D., Ferraro G.B., Jain R.K. (2020). The Blood–Brain Barrier and Blood–Tumour Barrier in Brain Tumours and Metastases. Nat. Rev. Cancer.

[17] Hajal C., Offeddu G.S., Shin Y., Zhang S., Morozova O., Hickman D., Knutson C.G., Kamm R.D. (2022). Engineered Human Blood–Brain Barrier Microfluidic Model for Vascular Permeability Analyses. Nat. Protoc..

[18] Yang G., Phua S.Z.F., Bindra A.K., Zhao Y. (2019). Degradability and Clearance of Inorganic Nanoparticles for Biomedical Applications. Adv. Mater..

[19] Ji D.-K., Reina G., Guo S., Eredia M., Samorì P., Ménard-Moyon C., Bianco A. (2020). Controlled Functionalization of Carbon Nanodots for Targeted Intracellular Production of Reactive Oxygen Species. Nanoscale Horiz..

[20] Simonneau C., Duschmalé M., Gavrilov A., Brandenberg N., Hoehnel S., Ceroni C., Lassalle E., Kassianidou E., Knoetgen H., Niewoehner J. (2021). Investigating Receptor-Mediated Antibody Transcytosis Using Blood–Brain Barrier Organoid Arrays. Fluids Barriers CNS.

[21] Tashima T. (2020). Smart Strategies for Therapeutic Agent Delivery into Brain across the Blood–Brain Barrier Using Receptor-Mediated Transcytosis. Chem. Pharm. Bull..

[22] Yuan Z., Wang B., Teng Y., Ho W., Hu B., Boakye-Yiadom K.O., Xu X., Zhang X.Q. (2022). Rational Design of Engineered H-Ferritin Nanoparticles with Improved siRNA Delivery Efficacy across an In Vitro Model of the Mouse BBB. Nanoscale.

[23] Shi X., Wang Z., Ren W., Chen L., Xu C., Li M., Fan S., Xu Y., Chen M., Zheng F. (2022). LDL Receptor-Related Protein 1 (LRP1), a Novel Target for Opening the Blood-Labyrinth Barrier (BLB). Signal Transduct. Target. Ther..

[24] Benitez-Amaro A., Pallara C., Nasarre L., Rivas-Urbina A., Benitez S., Vea A., Bornachea O., de Gonzalo-Calvo D., Serra-Mir G., Villegas S. (2019). Molecular Basis for the Protective Effects of Low-Density Lipoprotein Receptor-Related Protein 1 (LRP1)-Derived Peptides against LDL Aggregation. Biochim. Biophys. Acta Biomembr..

[25] Yamada K., Hashimoto T., Yabuki C., Nagae Y., Tachikawa M., Strickland D.K., Liu Q., Bu G., Basak J.M., Holtzman D.M. (2008). The Low Density Lipoprotein Receptor-Related Protein 1 Mediates Uptake of Amyloid β Peptides in an In Vitro Model of the Blood-Brain Barrier Cells. J. Biol. Chem..

[26] Terstappen G.C., Meyer A.H., Bell R.D., Zhang W. (2021). Strategies for Delivering Therapeutics across the Blood–Brain Barrier. Nat. Rev. Drug Discov..

[27] Hartl N., Adams F., Merkel O.M. (2021). From Adsorption to Covalent Bonding: Apolipoprotein E Functionalization of Polymeric Nanoparticles for Drug Delivery Across the Blood–Brain Barrier. Adv. Ther..

[28] Kim H.S., Lee S.J., Lee D.Y. (2022). Milk Protein-Shelled Gold Nanoparticles with Gastrointestinally Active Absorption for Aurotherapy to Brain Tumor. Bioact. Mater..

[29] Habib S., Singh M. (2022). Angiopep-2-Modified Nanoparticles for Brain-Directed Delivery of Therapeutics: A Review. Polymers.

[30] Sakamoto K., Shinohara T., Adachi Y., Asami T., Ohtaki T. (2017). A Novel LRP1-Binding Peptide L57 That Crosses the Blood Brain Barrier. Biochem. Biophys. Rep..

[31] Shi Y., Lammers T., Storm G., Hennink W.E. (2017). Physico-Chemical Strategies to Enhance Stability and Drug Retention of Polymeric Micelles for Tumor-Targeted Drug Delivery. Macromol. Biosci..

[32] Ghezzi M., Pescina S., Padula C., Santi P., Del Favero E., Cantù L., Nicoli S. (2021). Polymeric Micelles in Drug Delivery: An Insight of the Techniques for Their Characterization and Assessment in Biorelevant Conditions. J. Control. Release.

[33] Novo L., Rizzo L.Y., Golombek S.K., Dakwar G.R., Lou B., Remaut K., Mastrobattista E., Van Nostrum C.F., Jahnen-Dechent W., Kiessling F. (2014). Decationized Polyplexes as Stable and Safe Carrier Systems for Improved Biodistribution in Systemic Gene Therapy. J. Control. Release.

[34] Röder R., Helma J., Preiß T., Rädler J.O., Leonhardt H., Wagner E. (2017). Intracellular Delivery of Nanobodies for Imaging of Target Proteins in Live Cells. Pharm. Res..

[35] Leng Q., Mixson A.J. (2005). Modified Branched Peptides with a Histidine-Rich Tail Enhance in Vitro Gene Transfection. Nucleic Acids Res..

[36] Salcher E.E., Kos P., Fröhlich T., Badgujar N., Scheible M., Wagner E. (2012). Sequence-Defined Four-Arm Oligo(Ethanamino)Amides for pDNA and siRNA Delivery: Impact of Building Blocks on Efficacy. J. Control. Release.

[37] Lächelt U., Kos P., Mickler F.M., Herrmann A., Salcher E.E., Rödl W., Badgujar N., Bräuchle C., Wagner E. (2014). Fine-Tuning of Proton Sponges by Precise Diaminoethanes and Histidines in pDNA Polyplexes. Nanomed. Nanotechnol. Biol. Med..

[38] Benli-Hoppe T. (2023). Cationic Carrier Supported Peptide-Based Nanosystems for Tumor Targeting. Ph.D. Thesis.

[39] Prades R., Oller-Salvia B., Schwarzmaier S.M., Selva J., Moros M., Balbi M., Grazú V., de La Fuente J.M., Egea G., Plesnila N. (2015). Applying the Retro-Enantio Approach to Obtain a Peptide Capable of Overcoming the Blood-Brain Barrier. Angew. Chem. Int. Ed..

[40] Li Y., Lei Y., Wagner E., Xie C., Lu W., Zhu J., Shen J., Wang J., Liu M. (2013). Potent Retro-Inverso D-Peptide for Simultaneous Targeting of Angiogenic Blood Vasculature and Tumor Cells. Bioconjug. Chem..

[41] Wang J., Lei Y., Xie C., Lu W., Wagner E., Xie Z., Gao J., Zhang X., Yan Z., Liu M. (2014). Retro-Inverso CendR Peptide-Mediated Polyethyleneimine for Intracranial Glioblastoma-Targeting Gene Therapy. Bioconjug. Chem..

[42] Wei X., Zhan C., Chen X., Hou J., Xie C., Lu W. (2014). Retro-Inverso Isomer of Angiopep-2: A Stable D-Peptide Ligand Inspires Brain-Targeted Drug Delivery. Mol. Pharm..

[43] Sun Y., Zhang Y., Gao Y., Wang P., He G., Blum N.T., Lin J., Liu Q., Wang X., Huang P. (2020). Six Birds with One Stone: Versatile Nanoporphyrin for Single-Laser-Triggered Synergistic Phototheranostics and Robust Immune Activation. Adv. Mater..

[44] Deng S., Li X., Liu S., Chen J., Li M., Yian Chew S., Leong K.W., Cheng D. (2020). Codelivery of CRISPR-Cas9 and Chlorin e6 for Spatially Controlled Tumor-Specific Gene Editing with Synergistic Drug Effects. Sci. Adv..

[45] Pei P., Sun C., Tao W., Li J., Yang X., Wang J. (2019). ROS-Sensitive Thioketal-Linked Polyphosphoester-Doxorubicin Conjugate for Precise Phototriggered Locoregional Chemotherapy. Biomaterials.

[46] Jiang Y., Yang W., Zhang J., Meng F., Zhong Z. (2018). Protein Toxin Chaperoned by LRP-1-Targeted Virus-Mimicking Vesicles Induces High-Efficiency Glioblastoma Therapy In Vivo. Adv. Mater..

[47] Li L., Xi W.S., Su Q., Li Y., Yan G.H., Liu Y., Wang H., Cao A. (2019). Unexpected Size Effect: The Interplay between Different-Sized Nanoparticles in Their Cellular Uptake. Small.

[48] Donahue N.D., Acar H., Wilhelm S. (2019). Concepts of Nanoparticle Cellular Uptake, Intracellular Trafficking, and Kinetics in Nanomedicine. Adv. Drug Deliv. Rev..

[49] Xu Y., Li C., Lu S., Wang Z., Liu S., Yu X., Li X., Sun Y. (2022). Construction of Emissive Ruthenium(II) Metallacycle over 1000 nm Wavelength for in Vivo Biomedical Applications. Nat. Commun..

[50] Fisher C., Beglova N., Blacklow S.C. (2006). Structure of an LDLR-RAP Complex Reveals a General Mode for Ligand Recognition by Lipoprotein Receptors. Mol. Cell.

[51] Tao L., Tian S., Zhang J., Liu Z., Robinson-McCarthy L., Miyashita S.I., Breault D.T., Gerhard R., Oottamasathien S., Whelan S.P.J. (2019). Sulfated Glycosaminoglycans and Low-Density Lipoprotein Receptor Contribute to Clostridium Difficile Toxin A Entry into Cells. Nat. Microbiol..

