# Supplementary material for: Receptor-Targeted Carbon Nanodot Delivery through Polymer Caging and Click Chemistry-Supported LRP1 Ligand Attachment
Source: Polymers (Basel). 2023 Oct 10;15(20):4039. doi: 10.3390/polym15204039 (PMC10609667; doi:10.3390/polym15204039)
Supplement: Supplementary file 1 [file polymers-15-04039-s001.zip › polymers-2638673-supplementary.pdf]

Synthesis of four-armed OAA and targeting ligand-PEG conjugates.

Further synthetic details can be found in the original Ph.D. Thesis of Benli-Hoppe, T [1].

Table S1. Source of protected amino acids used in peptide synthesis.

Amino acid name	Supplier
Fmoc-D-Asp(OtBu)-OH	Iris Biotech (Marktredewitz, Germany)
Fmoc-L-Asp(OtBu)-OH	Iris Biotech (Marktredewitz, Germany)
Fmoc-Gly-OH	Iris Biotech (Marktredewitz, Germany)
Fmoc-D-His(Trt)-OH	Iris Biotech (Marktredewitz, Germany)
Fmoc-L-His(Trt)-OH	Iris Biotech (Marktredewitz, Germany)
Fmoc-D-Ile-OH	Iris Biotech (Marktredewitz, Germany)
Fmoc-L-Ile-OH	Iris Biotech (Marktredewitz, Germany)
Fmoc-D-Leu-OH	Iris Biotech (Marktredewitz, Germany)
Fmoc-L-Leu-OH	Iris Biotech (Marktredewitz, Germany)
Fmoc-D-Lys(Boc)-OH	Iris Biotech (Marktredewitz, Germany)
Fmoc-L-Lys(Boc)-OH	Iris Biotech (Marktredewitz, Germany)
Fmoc-D-Phe-OH	Iris Biotech (Marktredewitz, Germany)
Fmoc-L-Phe-OH	Iris Biotech (Marktredewitz, Germany)
Fmoc-D-Pro-OH*H_2O	Iris Biotech (Marktredewitz, Germany)
Fmoc-L-Pro-OH*H_2O	Iris Biotech (Marktredewitz, Germany)
Fmoc-D-Ser(tBu)-OH	Iris Biotech (Marktredewitz, Germany)
Fmoc-L-Ser(tBu)-OH	Iris Biotech (Marktredewitz, Germany)
Fmoc-D-Thr(tBu)-OH	Iris Biotech (Marktredewitz, Germany)
Fmoc-L-Thr(tBu)-OH	Iris Biotech (Marktredewitz, Germany)
Fmoc-D-Trp(Boc)-OH	Iris Biotech (Marktredewitz, Germany)
Fmoc-L-Trp(Boc)-OH	Iris Biotech (Marktredewitz, Germany)
Fmoc-D-Tyr(tBu)-OH	Iris Biotech (Marktredewitz, Germany)
Fmoc-L-Tyr(tBu)-OH	Iris Biotech (Marktredewitz, Germany)

1658

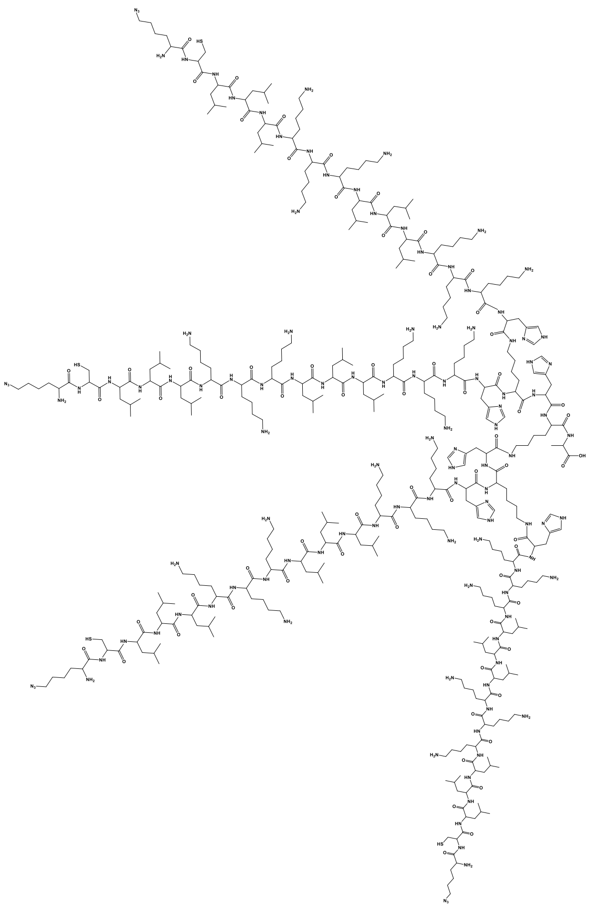

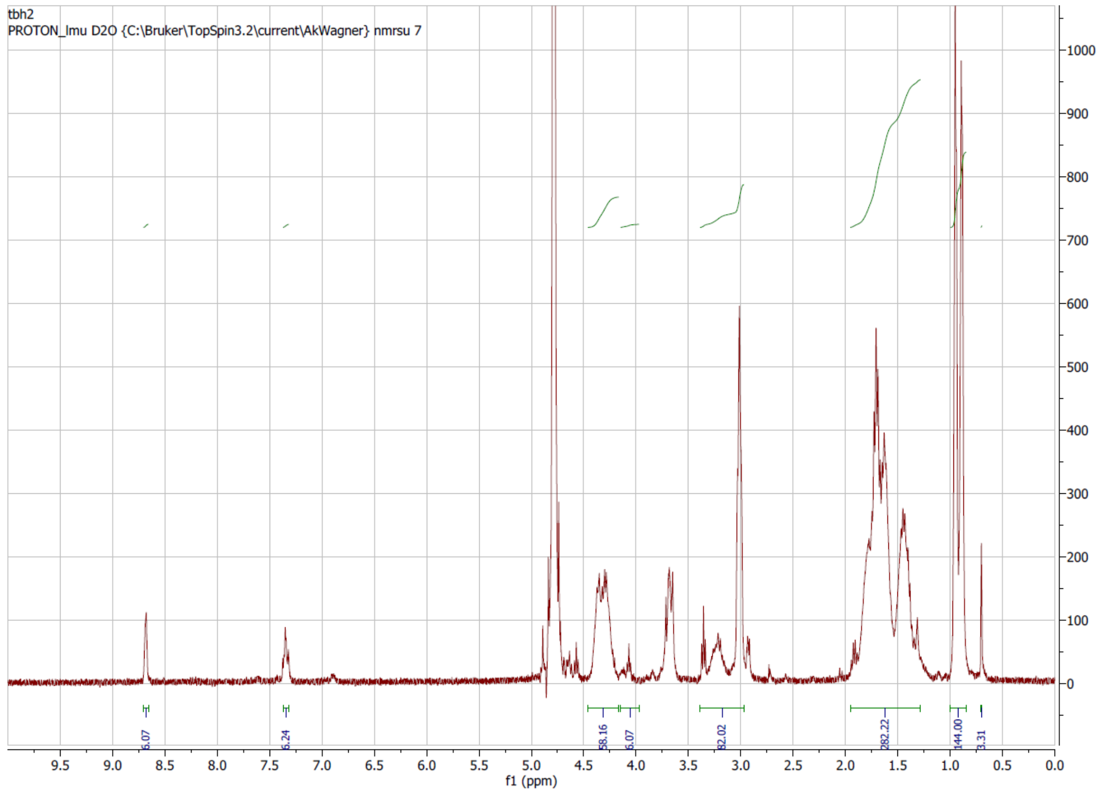
tbh2
PROTON_lmu D2O {C:\Bruker\TopSpin3.2\current\AkWagner} nmrsu 7
f1 (ppm)

Figure S1. Molecular structure and ^{1}H NMR spectrum of ***1658*** four-armed structure, recorded in D_2O. δ (ppm) = 0.5–0.7 (m, 3 H, βH alanine); 0.8–1.0 (m, 144 H, δH leucine); 1.3–1.9 (m, 282 H, βγδH lysine, βγ H leucine); 2.8–3.7 (m, 82 H, βH cysteine, βH histidine, εH lysine); 3.8–4.1 (m, 6 H, αH histidine); 4.2–4.4 (m, 60 H, αH alanine, αH cysteine, αH lysine, αH leucine); 7.6–7.7 (m, 6 H, imidazole); 8.4–8.5 (d, 6 H, imidazole).

1664

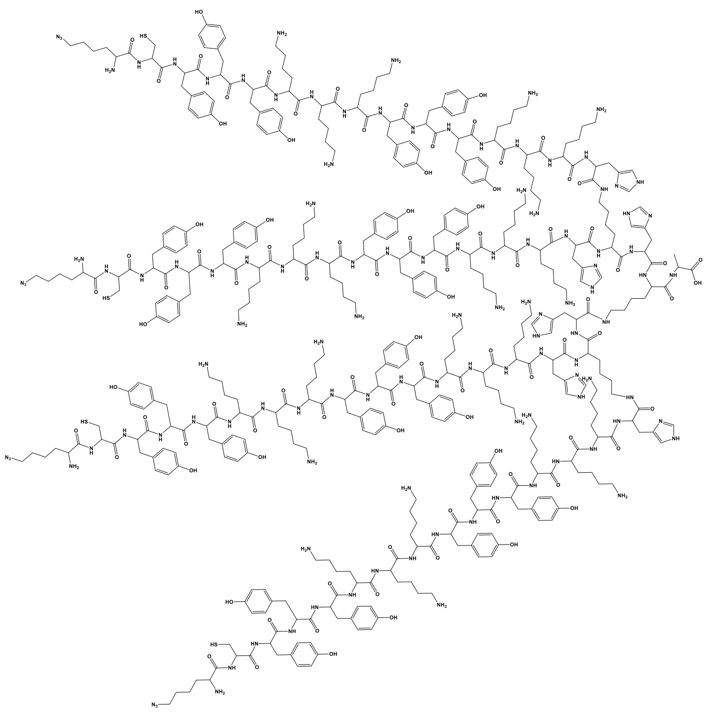

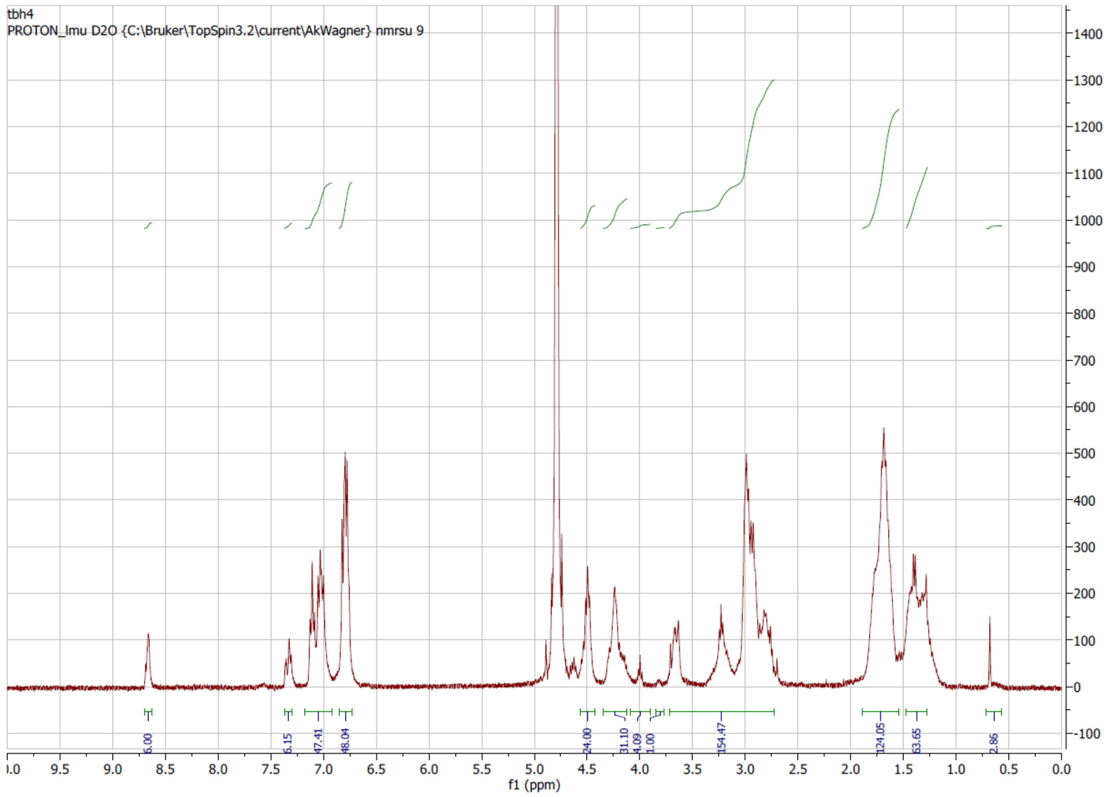

Figure S2. Molecular structure and ¹H NMR spectrum of ***1664*** four-arm structure, recorded in D_2O. δ (ppm) = 0.6–0.7 (m, 3 H, βH alanine); 1.25–1.45 (m, 62 H, γH lysine); 1.55–1.8 (m, 124 H, βδH lysine); 2.6–3.65 (m, 154 H, βH cysteine, βH histidine, εH lysine, αβH tyrosine); 3.75–4.1 (m, 5 H, αH cysteine, αH alanine); 4.1–4.4 (m, 31 H, αH lysine); 4.45–4.55 (m, 24 H, αH tyrosine); 6.7–6.8 (m, 48 H, phenol); 6.9–7.2 (d, 48 H, phenol); 7.3–7.4 (m, 6 H, imidazole);

8.6–8.7 (d, 6 H, imidazole).

1696

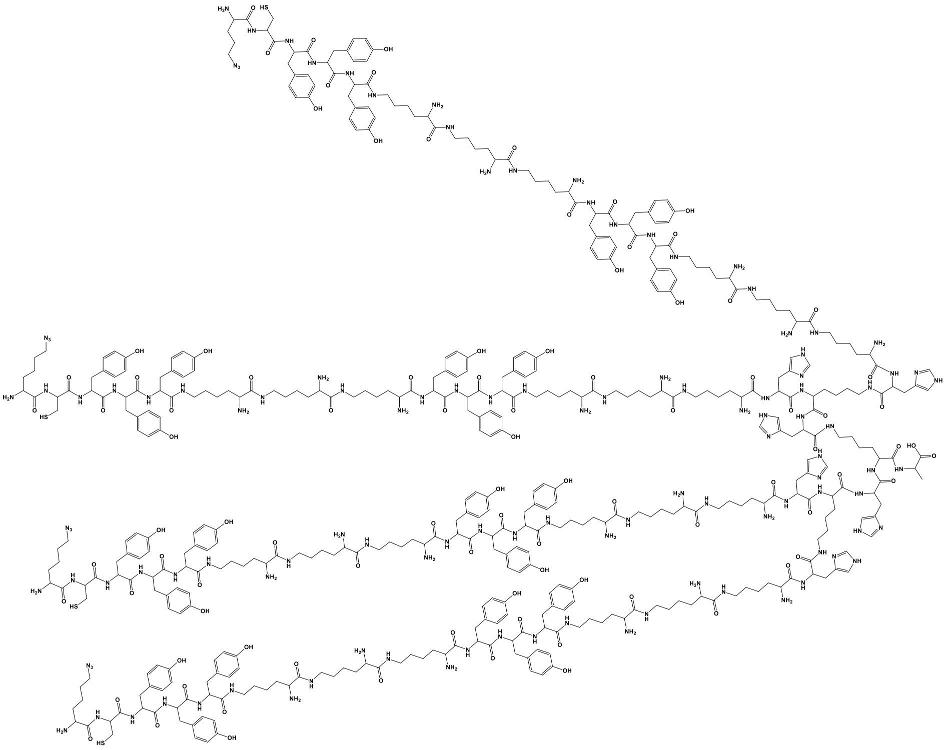

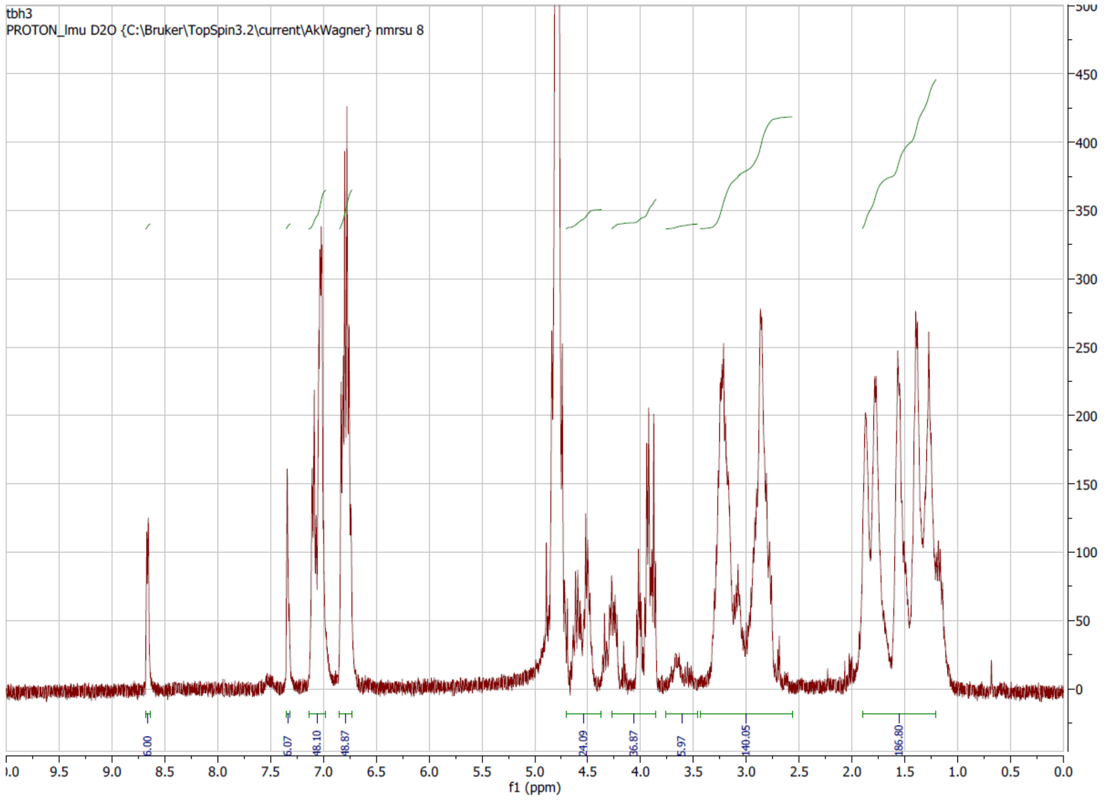

Figure S3. Molecular structure and ^{1}H NMR spectrum of ***1696*** four-arm structure, recorded in D_2O. δ (ppm) = 0.6–0.7 (m, 3 H, βH alanine); 1.2–1.85 (m, 186 H, γβδH lysine); 2.55–3.45 (m, 154 H, βH cysteine, βH histidine, εH lysine, αβH tyrosine); 3.5–3.6 (m, 5 H, αH cysteine, αH

alanine); 3.8–4.3 (m, 31 H, αH lysine); 4.4–4.6 (m, 24 H, αH tyrosine); 6.75–6.8 (m, 48 H, phenol); 6.9–7.2 (d, 48 H, phenol); 7.3–7.4 (m, 6 H, imidazole); 8.6–8.7 (d, 6 H, imidazole).

1768

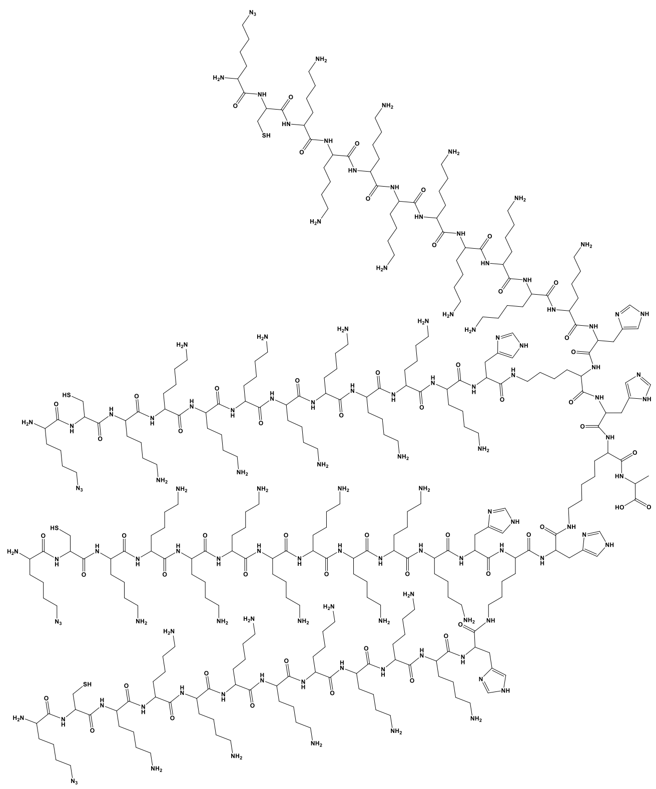

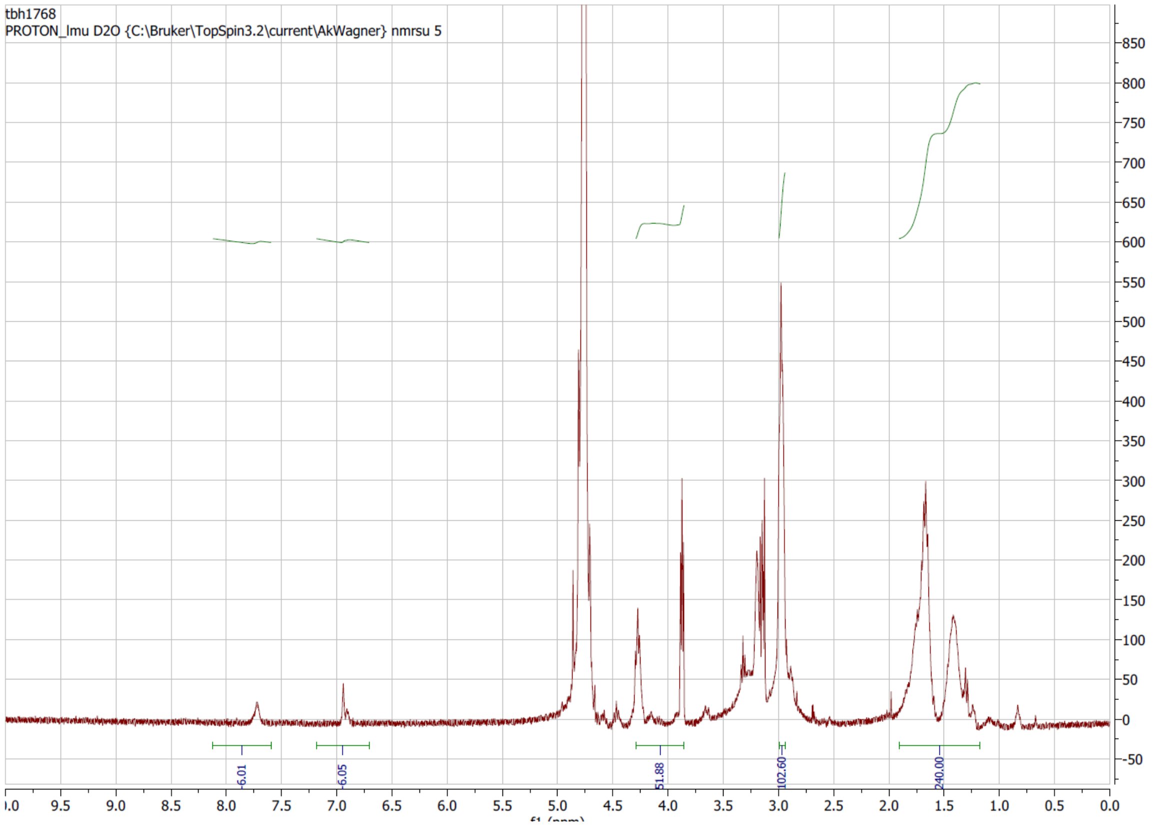

Figure S4. Molecular structure and ^{1}H NMR spectrum of ***1768*** four-arm structure, recorded in D_2O. δ (ppm) = 0.6–0.75 (m, 3 H, βH alanine); 1.2–1.8 (m, 240 H, γδβH lysine); 2.9–3.0 (m, 100 H, βH cysteine, βH histidine, εH lysine); 3.8–4.45 (m, 51 H, αH cysteine, αH alanine, αH

lysine, αH histidine); 6.7–7.2 (m, 6 H, imidazole); 7.6–8.1 (d, 6 H, imidazole).

1769

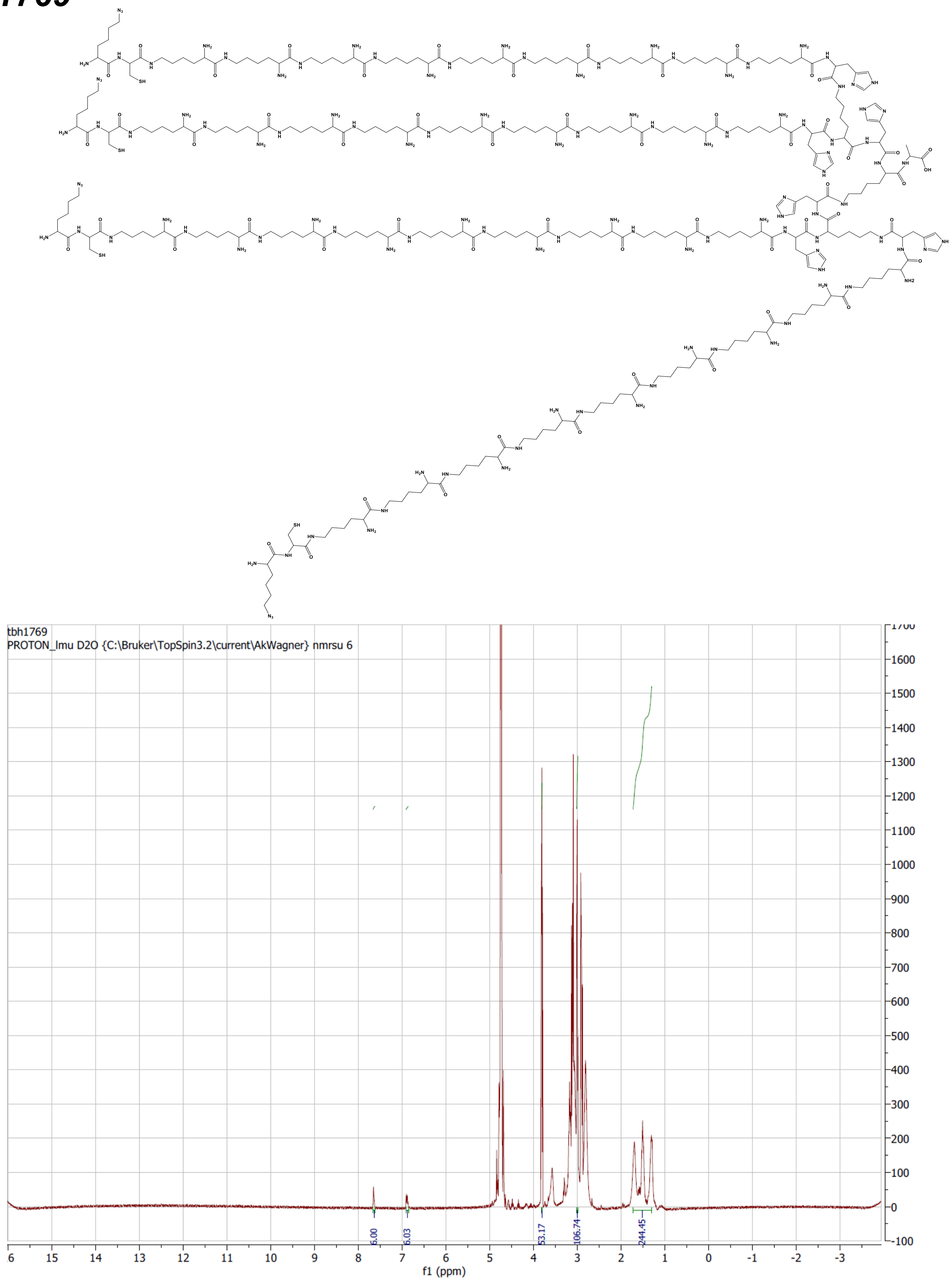

Figure S5. Molecular structure and ^{1}H NMR spectrum of ***1769*** four-arm structure, recorded in D_2O. δ (ppm) = 0.6–0.75 (m, 3 H, βH alanine); 1.2–1.7 (m, 240 H, γδβH lysine); 2.95–3.0 (m,

100 H, βH cysteine, βH histidine, εH lysine); 3.75–3.8 (m, 51 H, αH cysteine, αH alanine, αH lysine, αH histidine); 6.8–6.85 (m, 6 H, imidazole); 7.6–7.65 (d, 6 H, imidazole).

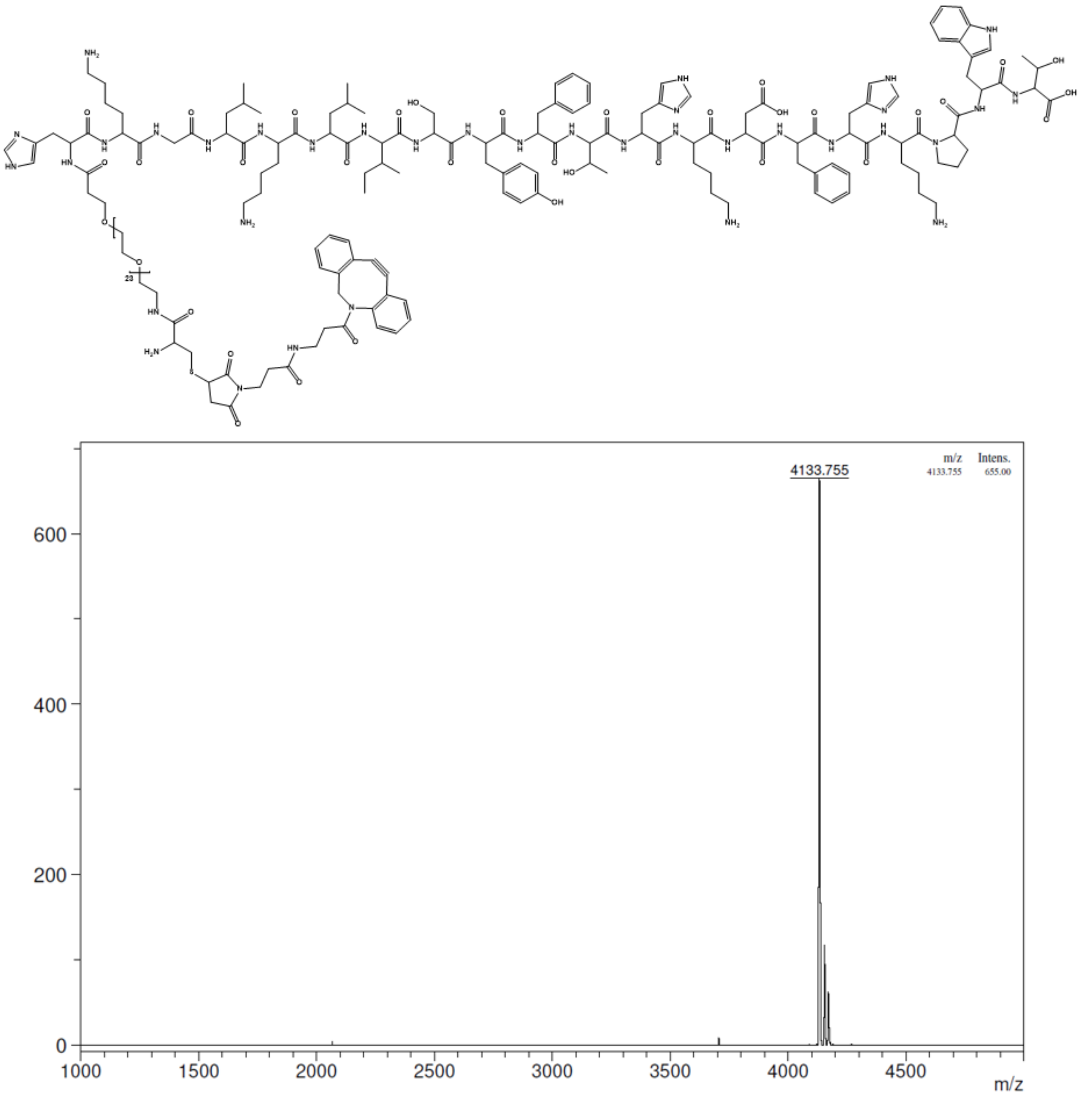

Figure S6. Molecular structure and MALDI-TOF-MS spectrum of reL57. $[M+H]^+$ calculated 4140. $[M+H]^+$ found 4134.

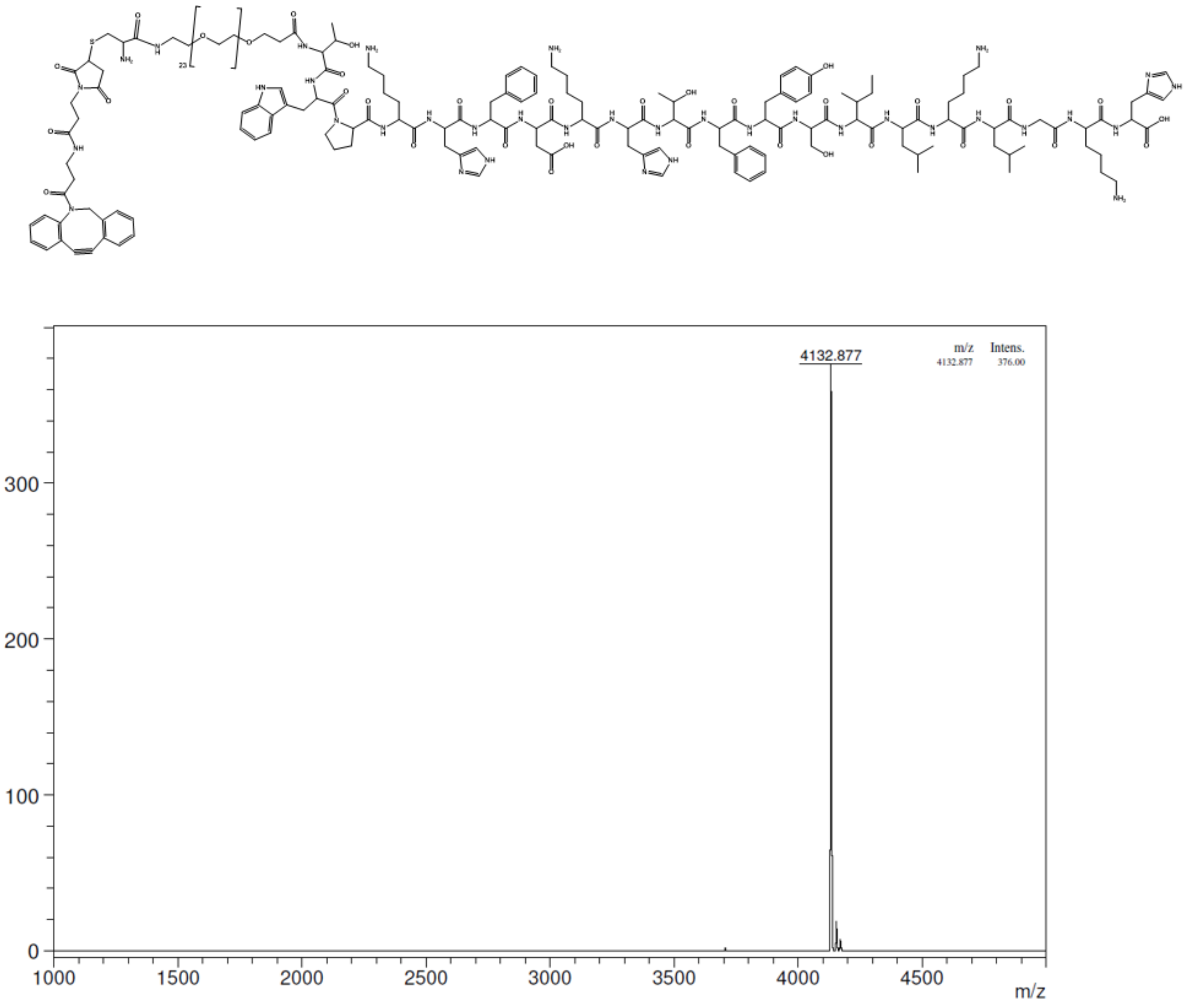

Figure S7. Molecular structure and MALDI-TOF-MS spectrum of L57. $[M+H]^+$ calculated 4140. $[M+H]^+$ found 4133.

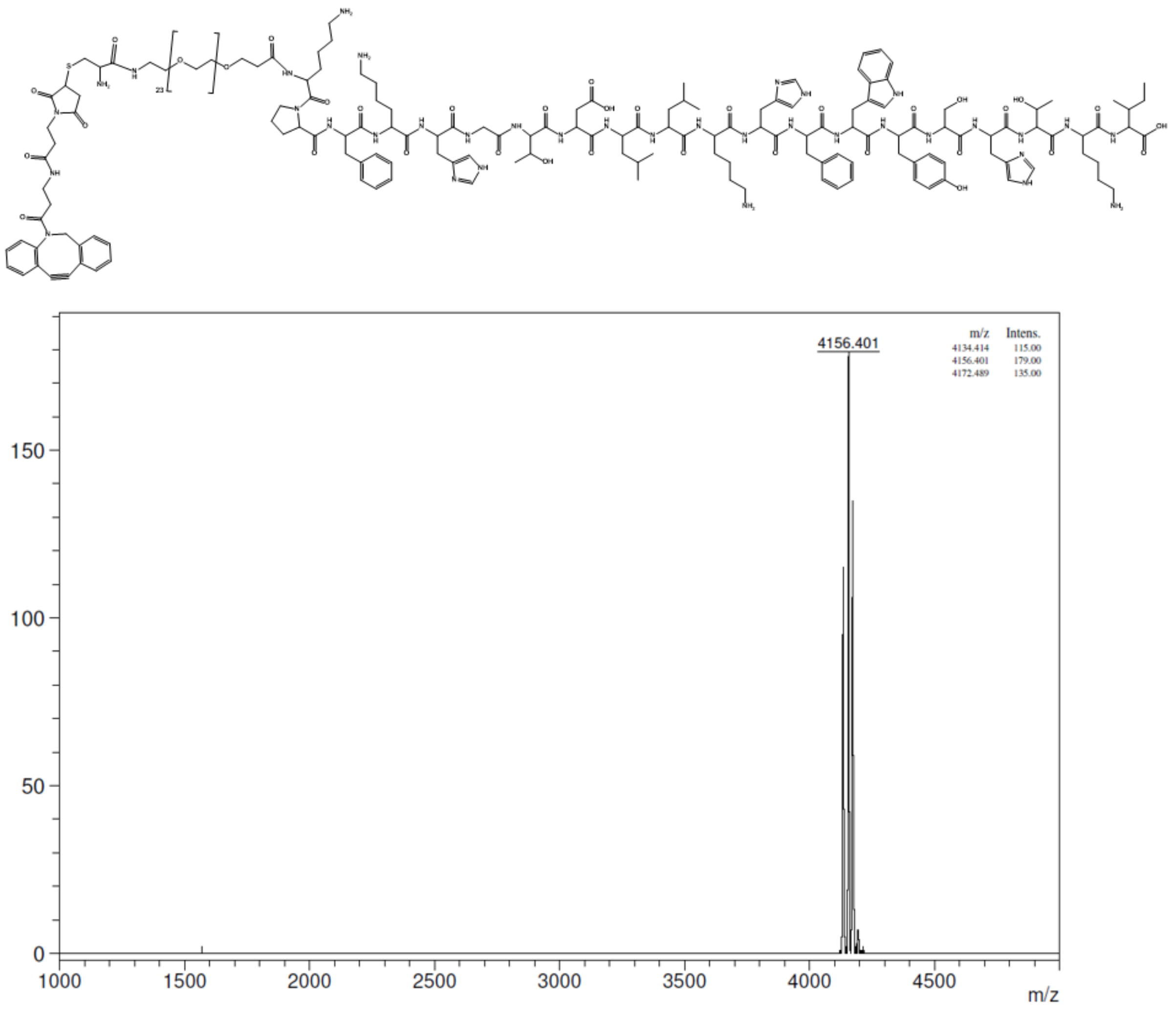

Figure S8. Molecular structure and MALDI-TOF-MS spectrum of scr-L57. $[M+H]^+$ calculated 4140. $[M+H]^+$ found 4134, $[M+Na]^+$ 4156, and $[M+K]^+$ 4172.

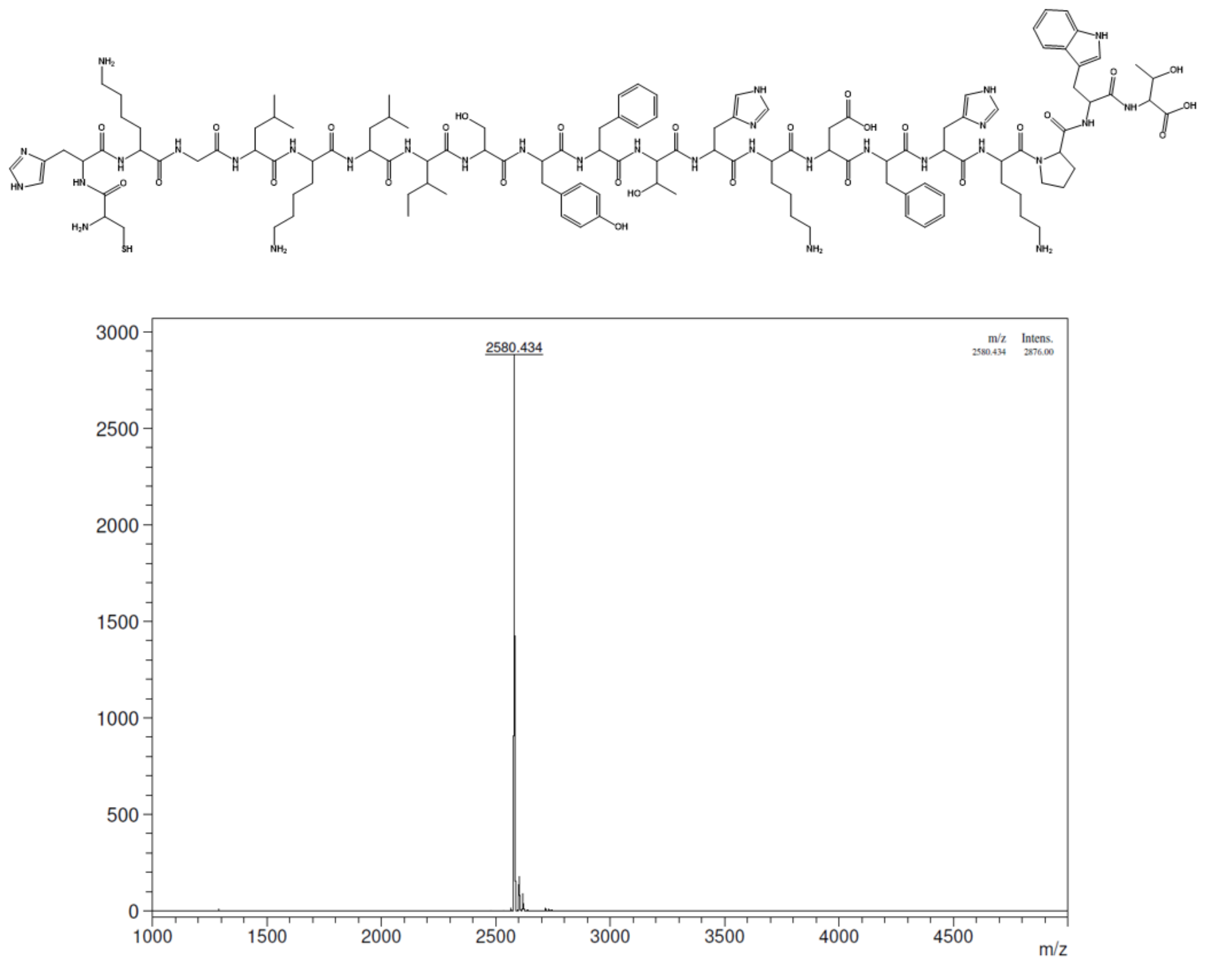

Figure S9. Molecular structure and MALDI-TOF-MS spectrum of re-L57-C. $[M+H]^+$ calculated 2585. $[M+H]^+$ found 2580.

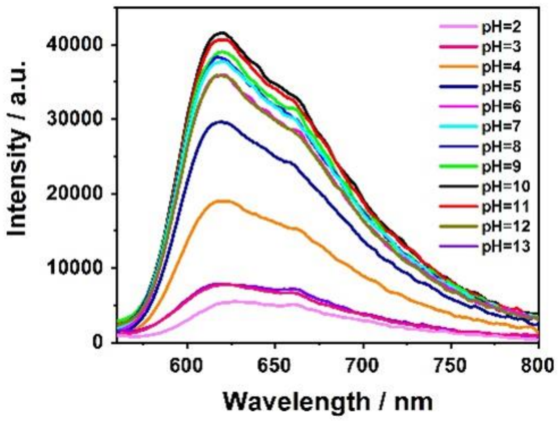

Figure S10. Fluorescence emission intensity of RCD aqueous solution under series pH conditions.

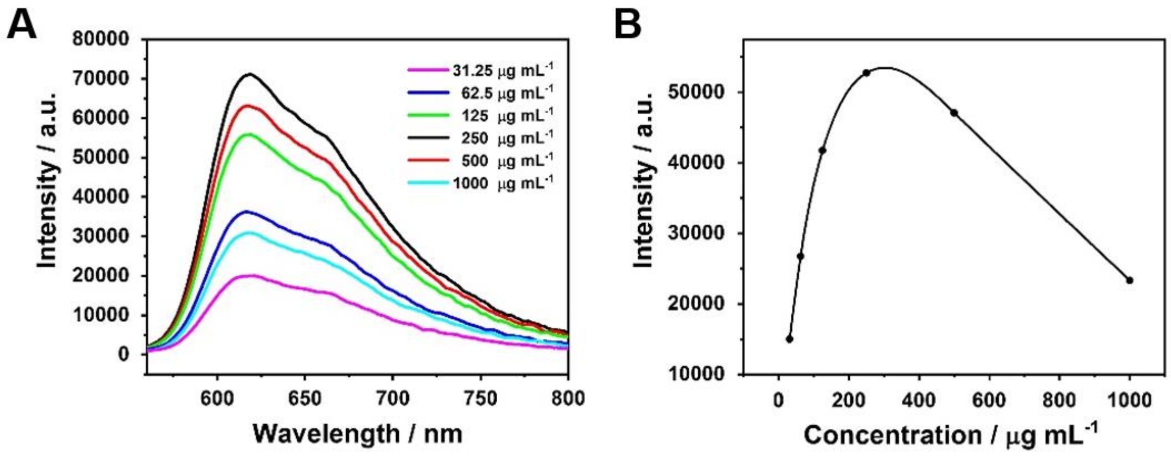

Figure S11. Fluorescence emission intensity of RCD aqueous solution with different concentrations.

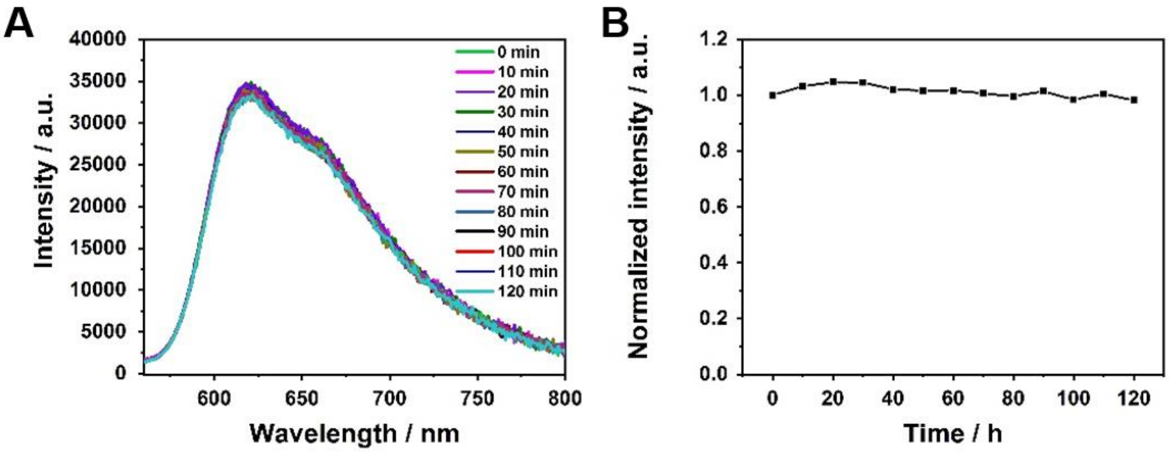

Figure S12. A) Fluorescence emission intensity and B) normalized fluorescence emission intensity of RCD aqueous solution (62.5 μg mL^{-1}) after irradiation for different times.

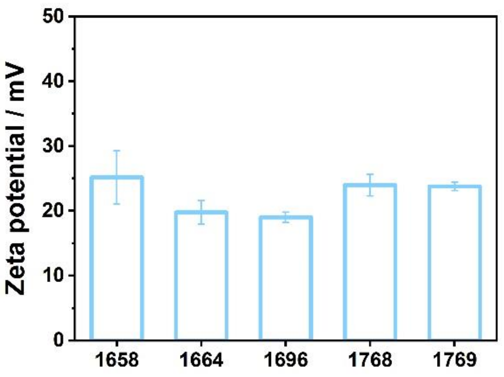

Figure S13. Zeta potential of RCD@OAA (***1658***, ***1664***, ***1696***, ***1768***, and ***1769***).

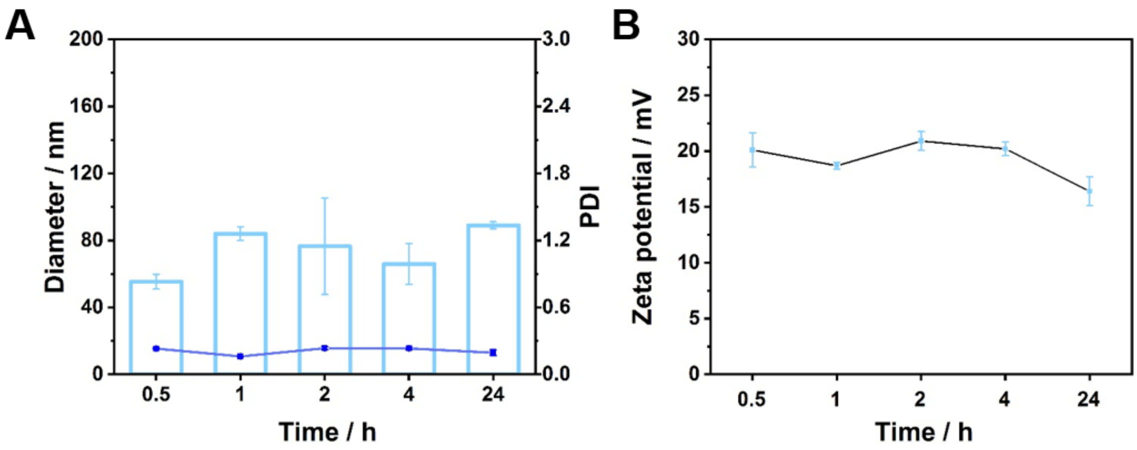

Figure S14. A) Hydrodynamic particle size (number), PDI, and B) zeta potential of RCD@1696 solution after incubation for different times.

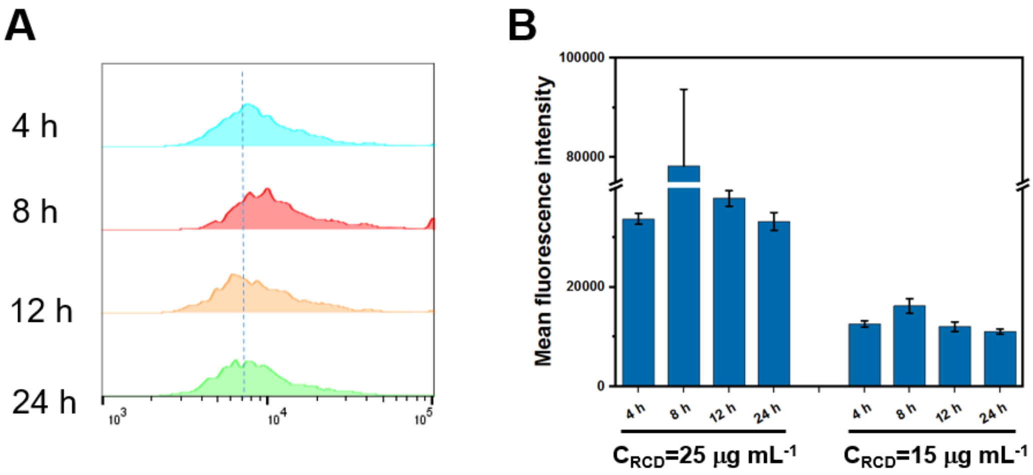

Figure S15. A) Cellular uptake assay of naked RCD (15 μg mL^{-1}) in U87MG cells measured by flow cytometry after 4 h, 8 h, 12 h, and 24 h incubation. B) The corresponding quantitative analysis of fluorescence intensity.

Reference

1. Benli-Hoppe, T. Cationic Carrier Supported Peptide-Based Nanosystems for Tumor Targeting, PhD Thesis, LMU Munich, Munich, 2023. doi: 10.5282/edoc.31733.
